# Care coordination gaps due to lack of interoperability in the United States: a qualitative study and literature review

**DOI:** 10.1186/s12913-016-1373-y

**Published:** 2016-04-22

**Authors:** Lipika Samal, Patricia C. Dykes, Jeffrey O. Greenberg, Omar Hasan, Arjun K. Venkatesh, Lynn A. Volk, David W. Bates

**Affiliations:** Division of General Internal Medicine and Primary Care, Brigham and Women’s Hospital, 1620 Tremont St., Suite OBC-03-02V, Boston, MA 02120 USA; Harvard Medical School, Boston, MA USA; American Medical Association, Chicago, IL USA; Yale University School of Medicine, New Haven, CT USA; Partners Healthcare System, Boston, MA USA

**Keywords:** Electronic health record, Meaningful use, Care coordination, Care transitions, Readmissions

## Abstract

**Background:**

Health information technology (HIT) could improve care coordination by providing clinicians remote access to information, improving legibility, and allowing asynchronous communication, among other mechanisms. We sought to determine, from a clinician perspective, how care is coordinated and to what extent HIT is involved when transitioning patients between emergency departments, acute care hospitals, skilled nursing facilities, and home health agencies in settings across the United States.

**Methods:**

We performed a qualitative study with clinicians and information technology professionals from six regions of the U.S. which were chosen as national leaders in HIT. We analyzed data through a two person consensus approach, assigning responses to each of nine care coordination activities. We also conducted a literature review of MEDLINE®, CINAHL®, and Embase, analyzing results of studies that examined interventions to improve information transfer during transitions of care.

**Results:**

We enrolled 29 respondents from 17 organizations and conducted six focus groups. Respondents reported how HIT is currently used for care coordination activities. HIT is currently used to monitor patients and to align systems-level resources with population needs. However, we identified multiple areas where the lack of interoperability leads to inefficient processes and missing data. Additionally, the literature review identified ten intervention studies that address information transfer, seven of which employed HIT and three of which utilized other communication methods such as telephone calls, faxed records, and nurse case management.

**Conclusions:**

Significant care coordination gaps exist due to the lack of interoperability across the United States. We must design, evaluate, and incentivize the use of HIT for care coordination. We should focus on the domains where we found the largest gaps: information transfer, systems to monitor patients, tools to support patients’ self-management goals, and tools to link patients and their caregivers with community resources.

**Electronic supplementary material:**

The online version of this article (doi:10.1186/s12913-016-1373-y) contains supplementary material, which is available to authorized users.

## Background

The goal of health information technology (HIT) is to improve the quality of healthcare and reduce healthcare costs, but evidence is mixed in the United States [1, 2]. One key strategy of the National Quality Forum (NQF) is to incorporate HIT into efforts to improve and measure care coordination [3]. We undertook a research investigation to inform the NQF’s “Critical Paths for Creating Data Platforms: Care Coordination Project,” a project with the ultimate goal of measuring care coordination on a national level using HIT.

As we have reported in a prior white paper, the literature on the use of HIT for care coordination only focuses on a few topics while care coordination literature in general focuses on nurse case management [4–6]. A topic which has recently come to the forefront in the US is interoperability [7]. Interoperability would allow information transfer during transitions and form a basis for many more opportunities for HIT to support care coordination. For example, when a patient sees multiple specialists these specialists need to negotiate responsibility for the patient’s medication regimen and other care [8, 9]. The lack of interoperability between the specialists’ electronic health records (EHRs) may be a barrier to development of new HIT tools to support this domain of care coordination.

We sought to determine the current state of HIT to support care coordination across multiple domains through two approaches: a qualitative study using ‘focus group-style interviews’ and a structured literature review. We sought to determine, from a clinician perspective, how care is coordinated and to what extent HIT is involved when transitioning patients between emergency departments (ED), acute care hospitals (ACH), skilled nursing facilities (SNF), and home health agencies (HHA) in settings across the United States.

## Methods

### Setting, participants, and interview structure

The NQF selected a Technical Expert Panel to represent a broad group of public and private stakeholders (Additional file 1). Based on the recommendations of this panel, we chose six regions of the country to represent geographic diversity and to include national leaders in HIT (Table 1). The sampling strategy was purposeful to identify study participants involved in clinical care, as well as information technology (IT) professionals with responsibility for transitional care. We employed a snowball methodology using our own contacts, contacts identified by the NQF, the Internet, and colleagues of the people that we contacted [10]. The clinicians were representative of different types of facilities. We were particularly interested in identifying clinicians on both sides of a care transition. For example, in Interview 1 we included a physician from a hospital and a physician from a post-acute nursing facility who had transferred patients to each other.

**Table 1:** Geographic region and care setting of respondents

Interview 1: University health system in Midwest region, respondents from acute care hospital (ACH) and skilled nursing facility (SNF)
Interview 2: National healthcare company with hospital, nursing center, and rehabilitation divisions, respondents from IT and SNF in New England
Interview 3: Mid-Atlantic region, respondents from an emergency department (ED), an ACH and a home health agency (HHA)
Interview 4: Integrated delivery system in New England, respondents from SNF, ACH, and HHA
Interview 5: University pediatric department in Northwest region, respondents from an ED, ACH, and HHA
Interview 6: National integrated delivery system, respondents from IT, an ACH and HHA

We conducted six one-hour ‘focus group-style interviews’ with clinicians and IT professionals. Interviews were conducted by two co-investigators (LS or PCD) over the telephone following a semi-structured interview guide (see Additional file 1). We developed the guide on the basis of the biomedical literature, our previous experience conducting qualitative interviews, and our own experiences as physicians and nurses. The study was designated as exempt from informed consent by the Partners HealthCare Institutional Review Board.

### Data analysis

We chose to use a closed coding process. Codes were assigned from a priori domains within the Agency for Healthcare Research and Quality (AHRQ) Care Coordination Measurement Framework [3]. This model was chosen due to inclusion of broad approaches as well as nine specific care coordination activities. For ease of reporting, we have collapsed the nine care coordination activities into three levels which align with the AHRQ framework: provider-level, patient-level, and system-level (Table 2).

**Table 2:** Care coordination activities from the AHRQ measurement framework collapsed into three levels

Care coordination activities	Level
1) Establish Accountability or Negotiate Responsibility	Provider-level
2) Communicate	
a. Interpersonal communication	
b. Information transfer	
3) Facilitate transitions	
4) Assess needs and goals	Patient-level
5) Create a proactive plan of care	
6) Monitor, follow up, and respond to change	
7) Support self-management goals	
8) Link to community resources	System-level
9) Align resources with patient and population needs	

Verbatim transcriptions of interviews were entered into QSR NVivo for coding and analysis. The interviews were coded through a two-person consensus approach (LS and PCD). The two coders read each transcript multiple times and annotated it using NVIVO software. Conflicts were resolved through consensus between the two reviewers. In order to describe the extent to which HIT is used for care coordination, we identified the overlap of the broad approach ‘HIT-enabled Coordination’ with each of the nine care coordination activities. In other words, we are presenting the responses that we assigned the code for AHRQ broad approach ‘HIT-enabled Coordination’ as well as one of the nine care coordination activity codes.

### Literature review methods

In addition to the interviews, we conducted a review of the literature. Our objective was to identify intervention studies conducted to improve transfer of information during transitions of care, with a focus on HIT interventions. We searched MEDLINE®, CINAHL®, and Embase with no date restrictions. Search terms related to information exchange during care transitions were tailored to each database and combined with search terms related to HIT. For example, MeSH terms included *Continuity of Patient Care, Patient Discharge, Aftercare, Consultation and Referral, Patient Transfer, Transportation of Patients, and Medical Documentation*. A detailed description of the Literature Review Methods are included in Additional file 2.

## Results

We sent 56 recruitment emails to clinicians and information technology professionals from 17 organizations in six regions. Over 50 % of our invited respondents (*N* = 29) were successfully enrolled and participated in an interview. All interviews were conducted between May and June 2012. A sample of responses is presented in Tables 3, 4, and 5.

**Table 3:** Interview responses about provider-level coordination activities

	Coordination activity	Interview/respondent site	
Response 1	Establish accountability or negotiate responsibility	Interview 1/ACH 1	“We developed a web based care management and care planning tracking system. The nurse practitioner (NP) and social worker (SW) go in and identify protocols that apply to the particular individual… So at the team conference with the pharmacist, mental health, and geriatrician, they all provide input … the NP and SW then use that tool as an ongoing way to track implementation and the weekly team conference provides a kind of accountability and problem solving. If something’s not getting done, how come?”
Response 2	Interpersonal communication	Interview 1/ACH 2	“We have a very close network, so if I’m sending a person to [Doctor A] in house calls, I’ll shoot him an email or give him a page. And similarly, [Doctor B] and I often communicate and not only about the good stuff but if something went wrong we are very accountable to each other and let each other know ‘this didn’t go as smoothly as it might have seemed,’ and that way we can always hope to better our programs for patient care.”
Response 3	Information transfer	Interview 1/SNF	“For patients who are coming from Hospital A and Hospital B, we do have a computer available in at least a couple of our facilities where we can log in and really extract information from the medical records. It is very time consuming, logging in some days is not that great or internet issues and all that… But I know my nurse practitioner regularly logs onto the computer and tries to extract important pieces of information. In terms of getting discharge summaries, it’s still a huge challenge. I would say that with [Hospital C] I only receive discharge summaries on probably 50 % of the patients. They tried to improve this and, even though the residents are doing them before the patient leaves, getting them on the ambulance with the patient just does not work out all the time.”
Response 4	Information transfer	Interview 2/IT	“[The pre-admission clinical evaluation] is captured electronically, but it’s sent as a pdf. It supports what’s affectionately sometimes called the swivel chair interface, you can swivel your chair from one screen to another screen as you read key stuff. So it’s not an ideal interface, but it’s also a very controlled interface. What we’ve had in the past when we’ve tried to just plug different systems together and taken some data from some e-referral solutions is we get data quality problems when we bring the data in. They don’t have the name right, they don’t have the address right, they don’t have the date of birth right, they don’t have the payer right, they don’t have the payer ID right.”
Response 5	Information transfer	Interview 2/SNF	“If information is missing when the patient comes in to the LPAC, we typically will go to our clinical liaison and ask them to get us the missing pieces from the short term acute care hospital. We’ve had discharge summaries missing or pieces of a medication record missing or a health care proxy, things like that. But we’ll typically reach right back out to that clinical liaison who has a relationship with the short term acute care hospital, and get that for us as soon as possible.”
Response 6	Facilitate transitions	Interview 3/HHA	“We go and look in a variety of systems: the system that most of the hospital discharge planners are using, our medication administration and order entry system, and we can also look in our outpatient system… you end up having clinical people, nurses doing a lot of clerical work because, how do you divide that workflow up? They’re the one combing through the chart to find it.”

*ACH* acute care hospital, *SNF* skilled nursing facility, *LPAC* long-term post acute care, *HHA* home health agency

**Table 4:** Interview responses about patient-level coordination activities

	Coordination activity	Interview/respondent site	
Response 7	Assess needs and goals	Interview 6/ACH	“In addition, there’s some functionalities in [our EHR]…What are the patient’s goals of care? and you can enter it into a field that is automatically pulled in. So it might say, ‘The patient wants to get to their son’s graduation,’ or, ‘They’re not ready to quit smoking, but they’re ready to do this,’ so we’re not asking the patient all the time.”
Response 8	Create a proactive plan of care	Interview 3/ACH	“Every day the patient gets an itinerary of exactly what will happen to them that day in their plan of care and a spot to write their questions and their concerns about what’s happening and that is addressed during those care coordination rounds every day.”
Response 9	Monitor, follow up, and respond to change	Interview 5/ACH	“We have a certain e-mail trigger that, when any of our patients who are identified with medically complex child service, anytime they hit the institution, there’s an automatic e-mail sent to that inbox.”
Response 10	Support self-management goals	Interview 4/HHA	“We’re also doing chronic care management training with our clinicians. That has a lot of things like telephone triaging, really looking at the patient and determining their specific goals. One of their goals may be to stay out of the hospital. There’s a lot of those things, however none of it is really software driven, meaning the software doesn’t have the logic to help with the decision making to help the clinician with any specific care plan or interventions or anything like that.”

*ACH* acute care hospital, *HHA* home health agency

**Table 5:** Interview responses about system-level coordination activities

	Care coordination activity	Interview/respondent site	
Response 11	Link to community resources	Interview 1/SNF	“It doesn’t give any information about the services that they provide or the quality. And unfortunately that’s how these things stand, and most of these suggestions are being made based on the patient’s distance from the family and not much thought is being put into it. That’s the standard practice. There are some hospitals which are now trying to use nursing home compare websites, which has the benefit that it has the star ratings and some quality markers on it.”
Response 12	Align resources with patient and population needs	Interview 6/ACH	“We build queues or use questionnaire functionalities within the EHR and then we can routinely get data back on it. So for example, what interventions did the nurse do in terms of care coordination? So we know that the primary intervention the nurse making the call has to do is medication and navigation. So did the patient get their appointment, their meds? There are about ten potential interventions but those are the top two, just to give you some examples.”

### Interview responses pertaining to provider-level care coordination activities

‘Establishing accountability’ concerns interpersonal relationships and is a process that is governed by local policies and customs. HIT was used for this care coordination activity, as described in Interview 1 (Table 3, Response 1). In this instance, the negotiation of responsibility occurs in a face-to-face meeting, but clinician accountability was tracked using a web-based tool.

The AHRQ framework distinguishes between two types of Communication: Interpersonal communication and Information transfer [3]. We did see that email was used for interpersonal communication, which allows hand-offs to be performed asynchronously (Table 3, Response 2). However, due to the lack of interoperability, none of the regions reported electronic information transfer. Instead, medical records were printed out and hand-carried by ambulance personnel (Table 3, Response 3). The ED and acute care hospitals in interview 6 shared the same EHR, but they lacked interoperability with SNF and HHA organizations. We asked about effectiveness of information transfer and how clinicians respond to missing information and found that significant care coordination gaps exist due to the lack of interoperability (Table 3, Responses 3, 4 and 5).

There were no examples of innovative HIT tools to facilitate transitions. We did note that there were many organizations that have hired nurse case managers to facilitate transitions between acute care hospitals and EDs. Organizations in interviews 1 and 3 use a case manager and social worker to integrate care between inpatient and outpatient teams. Long-term acute care hospitals in interview 2 used nurses in a similar role. EDs in interviews 3, 4, and 5 employed nurses to make follow-up appointments and arrange transfer back to sub-acute facilities. As a nurse from a home health agency in nterview 3 explained, the lack of interoperability forces clinicians to do clerical work (Table 3, Response 6).

In summary, HIT was occasionally used in each of the provider-level care coordination activities, but the processes were inefficient due to the lack of interoperability. Interoperability would spur innovative HIT tools to assist nurses and physicians while they are searching for missing information.

### Interview responses pertaining to patient-level care coordination activities

HIT was used to assess patients’ needs and goals. ACH respondents from Interviews 3 and 6 spoke about an EHR patient portal that included shared decision-making tools and a place to enter patient goals (Table 4, Response 7).

Clinicians were not able to create a proactive plan of care electronically, though an ACH respondent in Interview 3 mentioned that a hospital in their network was using an inpatient EHR to help patients understand their own plan of care (Table 4, Response 8). We have published a separate paper about the current and future state of longitudinal care plans, where we reported that these plans were not truly longitudinal since they exist in one setting and were not carried forward with the patient [11].

There were several electronic tools to support the activity ‘Monitor, follow-up and respond to change,’ which is the process by which clinicians respond to changes in clinical status for an individual patient. One site had a standardized note template in the EHR for post-discharge phone calls, another had a tele-management program for congestive heart failure and another had an email trigger that automatically sent an email when a patient from the medically complex child service was admitted anywhere within the system (Table 4, Response 9).

There were only a few examples of the use of HIT to support patients’ self-management goals. Three regions utilized a patient portal where patients could view their own medication lists, though clinicians thought that the information should be tailored to patient literacy level. One respondent from a home health agency pointed out that the software that they were using was not sophisticated enough to help clinicians with individualized support for patients’ goals (Table 4, Response 10).

In summary, patient portals were used to support patient-level care coordination and case management programs were in place to follow-up with patients. There were few examples of HIT tools that clinicians and patients could use together to tailor management. There were no examples of free-standing patient HIT tools which were interoperable with EHRs.

### Interview responses pertaining to system-level care coordination activities

One of the activities that should be addressed at a system-level is ‘Link to community resources,’ which are defined by AHRQ as, “any service or program outside the health care system that may support a patient's health and wellness (e.g., Medicaid, food stamps, social services, educational resources, support groups, or Meals on Wheels).” The only electronic tool for this activity was cited by respondents from a SNF in interview 1 who described an Internet directory of nursing facilities, but focused on the shortcomings of the system (Table 5, Response 11).

The ninth care coordination activity is ‘Align resources with patient and population needs.’ Many respondents use risk stratification tools to identify the sickest patients. An HHA respondent in Interview 4 described a paper tool completed in the patient’s home as an education tool to highlight factors such as poly-pharmacy, chronic diseases, and multiple hospitalizations. A respondent from a SNF in Interview 2 described an electronic tool from a commercial EHR company that helps to identify patients at high risk according to their symptoms. Interview 3 HHA gathers data electronically on the number of admissions, medications, and chronic conditions to develop a risk score; patients with high scores have two ‘front-loaded’ home visits soon after discharge. The Interview 6 hospital explained that they use a validated tool that has been integrated into the EHR, called LACE, which is an acronym for Length of stay, Acute admissions through the ED, Co-morbidities, and ED visits in the past 6 months [12]. An ED respondent from Interview 3 described an EHR alert for patients with three ED visits or a readmission in the past 30 days. An ACH respondent in Interview 6 described sophisticated EHR tools that incorporate patient-specific data (Table 5, Response 12).

In summary, there were no examples of interoperability between an EHR and a computer system used by a community organization. There were many examples of HIT tools to align system-level resources with high-risk patients or populations.

### Literature review results

Our literature search initially identified 173 citations. Each citation was reviewed by two reviewers and conflicts were resolved by consensus. After title-abstract review, 54 articles were included for full article review by two reviewers. Of these articles, 44 were excluded prior to data extraction due to the fact that they were not studies of interventions to improve information exchange during care transitions (see Additional file 2). The ten remaining articles were abstracted according to a standard format. Three of the articles described non-HIT interventions to improve information transfer across settings [13–15]. We found seven articles which described the use of HIT for information transfer across care transitions [16–22]. None of these interventions leveraged interoperable computer systems. A detailed summary of the results is included in Additional file 2.

## Discussion

We have found few intervention studies of HIT-supported care coordination in the biomedical literature and the results of our qualitative study show that, while HIT is used for several care coordination activities, there are important gaps. Even though we found high adoption of EHRs in acute care hospitals and EDs, as well as several SNFs and HHAs, these EHRs are not interoperable, that is they are not able to send and receive information electronically.

Studies from Europe show the advantages of HIT-supported care coordination [23, 24]. In order to realize these advantages in the US, we must identify the care coordination domains with the highest potential for HIT. Our approach identifies weaknesses in processes of care coordination, which are variably amenable to improvement in HIT enablement. Not all processes of care coordination can or should be automated. For example, ‘Establishing Accountability and negotiating responsibility’ has low potential for HIT because clinician roles and responsibilities must be defined by standard policies and interpersonal relationships [25]. We present our interpretation of the unrealized potential for HIT in each of the nine AHRQ care coordination domains through a side-by-side comparison of current capabilities of HIT (Table 6, Column I) and the potential for HIT (Table 6, Column II). In addition to the provider-level domains which could be improved through EHR interoperability, there are several patient-level care coordination domains which could be improved through other types of HIT innovation. For example, ‘Creating a Proactive Plan of Care’ could be improved through electronic longitudinal care plans [11].

**Table 6:** Interpretation of gaps between current capability of HIT and future potential for HIT to support care coordination

	I.	II.
AHRQ care coordination activities	Current capability of HIT	Future potential for HIT^a^
Establish accountability or negotiate responsibility	0	Low
Communicate		
Interpersonal communication	+	Low
Information transfer	0	High
Facilitate transitions	0	Moderate
Assess needs and goals	++	Moderate
Create a proactive plan of care	0	Moderate
Monitor, follow up, and respond to change	+	High
Support self-management goals	0	High
Link to community resources	+	High
Align resources with patient and population needs	++	High

^a^‘Low’ potential indicates that HIT has a limited role. ‘Moderate’ potential indicates that HIT could an instrumental support for people and processes. ‘High’ potential indicates that the care coordination activity could be almost completely automated with oversight by clinicians

In addition to care coordination gaps related to the lack of interoperability, we also identified important gaps in communication with community organizations. As one study showed, a missing ‘Emergency Contact’ in the EHR is predictive of readmissions [26]. Therefore, HIT tools that link patients to community resources are needed to prevent readmissions. One possibility would be to leverage the information which is stored in the clinical record, such as the patient’s zip code, to automatically enroll patients in community programs upon discharge. Interoperability between EHRs and community organizations’ computer systems would also be advantageous. Even giving community organizations the ability to view patients’ upcoming appointments, referral information, medications and medical history would be useful [27].

This qualitative study provides primary data about care coordination gaps across multiple regions, in diverse clinical settings, and across the disciplines of nursing and medicine. Despite these strengths, the study has several important limitations including a purposive sampling approach of innovative health systems in a small number of settings, limiting our ability to comment generally on the situation across the nation. Also, we developed the interview guide to explore the use of HIT for care coordination quality measurement specifically. The main limitation of the literature review is that the search terms in all three databases do not accurately identify articles related to care transitions. Despite these limitations, we found qualitative evidence that HIT is used for nine care coordination activities and we found ten intervention studies that yielded useful information about the impact of care coordination interventions on clinical outcomes.

## Conclusions

The intent of the Health Information Technology for Economic and Clinical Health (HITECH) Act was to improve the quality, safety and cost of health care through HIT, but we have not yet achieved these expected benefits [1, 28]. Our collective focus on reducing readmissions has spurred innovation in HIT development to align resources with patient and population needs. More innovation is needed and expected by the public, especially in the domains where we found the largest gaps: information transfer, systems to monitor patients, tools to support patients’ self-management goals, and tools to link patients and their caregivers with community resources. The key barrier to effective HIT interventions is the lack of interoperability between EHRs, patient HIT tools, and community organizations’ HIT tools. As we design future stages of Meaningful Use and other HIT policy, we must rigorously evaluate the impact of HIT on care coordination and incentivize widespread adoption of effective HIT tools.

### Availability of data and materials

The dataset supporting the conclusions of this article is available upon request as a 105 page pdf document. The dataset is not available as an electronic document because NVIVO file containing the two reviewers’ annotations became corrupted due to improper syncing between the hard drive and secure shared drive.

## Additional files

Additional file 1: A file containing the interview guide. (DOCX 15.4 kb) Additional file 2: A file containing the literature review methods and results. (DOCX 77.2 kb)

## Abbreviations

ACH: acute care hospital
AHRQ: Agency for Healthcare Research and Quality
ED: emergency department
EHR: electronic health record
HHA: home health agency
HIT: Health Information Technology
NQF: National Quality Forum
SNF: skilled nursing facility
