# Supplementary material for: Care coordination gaps due to lack of interoperability in the United States: a qualitative study and literature review
Source: BMC Health Serv Res. 2016 Apr 22;16:143. doi: 10.1186/s12913-016-1373-y (PMC4841960; doi:10.1186/s12913-016-1373-y)
Supplement: Additional file 1: — A file containing the interview guide. (DOCX 15.4 kb) [file 12913_2016_1373_MOESM1_ESM.docx]

**Additional file 1**

**Technical Expert Panel Roster**

- Kathryn H Bowles, PhD, RN, FAAN, Associate Professor of Nursing, University of Pennsylvania School of Nursing, Philadelphia, PA
- Patricia Button, EdD, RN, Chief Nursing Officer, Zynx Health Incorporated, Los Angeles CA
- Maureen Dailey DNSc, RN, CWOCN, Senior Policy Fellow, National Center for Nursing Quality, American Nurses Association, Silver Spring, MD
- Laura Heermann Langford, RN, PhD, Director, Nursing Informatics, Intermountain Healthcare, Salt Lake City, UT
- Gerri Lamb, PhD, RN, FAAN, College of Nursing & Health Innovation, Arizona State University, Phoenix AZ
- Rita M. Mangione-Smith, MD, MPH, Professor of Pediatrics and Adjunct Professor of Health Services, Seattle Children's Research Institute, Seattle, WA
- Jeffrey Riggio, MD, MS, Medical Director, Clinical Informatics Thomas Jefferson University Hospital, Philadelphia, PA
- David A Stumpf, MD, PhD, Professor Emeritus, Northwestern University,
- Woodstock Health Information & Technology, Woodstock, IL
- Judith Tobin, PT, MBA, Technical Adviser, Centers for Medicare & Medicaid Services, Office of Clinical Standards and Quality, Baltimore, MD
- Susan Yendro, RN, BSN, Associate Project Director, Department of Quality Measurement, The Joint Commission, Oakbrook, Terrace, IL

**Interview guide**

| Tell us about how you try to improve care coordination at your organizations (strategies and approaches). |
| --- |
| What structures do you have in place to coordinate care?  (Cue for the following: assigning patients to a PCP or patient-centered medical home before discharge and identifying high risk patients who need intensive care coordination) |
| What electronic tools are available to coordinate care?  (Cue for the following: electronic tracking tools within a care team, electronic information exchange, risk stratification tools, electronic tools for patient engagement) |
| Are there any other tools you think would be helpful? Probe for tools in other parts of their organization. |
| What tasks must be completed when there is missing information at the time of transfer? How do you obtain the missing information you need? Ultimately, who is responsible if information is missing? |
| How does your organization implement a longitudinal plan of care? |
| How is the longitudinal plan of care integrated with the care coordination tools described above? |
| How do you to track care coordination? (Probe for specific metrics.) |
| What else would you like us to know about how you plan for care transitions? |
