# Supplementary material for: Care coordination gaps due to lack of interoperability in the United States: a qualitative study and literature review
Source: BMC Health Serv Res. 2016 Apr 22;16:143. doi: 10.1186/s12913-016-1373-y (PMC4841960; doi:10.1186/s12913-016-1373-y)
Supplement: Additional file 2: — A file containing the literature review methods and results. (DOCX 77.2 kb) [file 12913_2016_1373_MOESM2_ESM.docx]

**Additional file 2**

Literature Review Methods

We included studies that examined the impact of interventions to improve information exchange during transitions of care. Each citation that was retrieved by the search was first reviewed by two reviewers in title-abstract format. The full text of included articles was retrieved and reviewed by two reviewers. We excluded qualitative studies, review articles, technical reports, and editorials, as well as descriptive studies utilizing chart reviews (e.g., chart reviews to identify deficiencies of discharge documents).

We utilized a standardized data collection instrument to extract pertinent data elements from each study including: study design, number of patients, population, setting, intervention, control condition, outcome measures, and results. We extracted outcomes such as: clinician perceptions of usefulness of care coordination interventions, rate of use of electronic tools for care coordination, effect on process outcomes (e.g., time to post discharge primary care visit, 30 day readmission rate, healthcare costs, length of stay), and other measures of quality.

**Figure 2. PRISMA diagram of articles excluded from the literature review**

Citations from CINAHL:

**n = 51**

Citations from EMBASE:

**n = 44**

Citations from Pubmed:

**n = 78**

Total abstracts retrieved:

**n = 173**

Unique abstracts retrieved:

**n = 160**

Exclusion:

**13 duplicates**

Abstracts excluded:

**n = 106**

Full-text articles retrieved:

**n = 54**

Articles included in review

**n = 10**

**Full-text articles excluded: 44**

**11** Editorials

**6**  Qualitative reports

**5** Identifying deficiencies via survey or chart review

**4** Literature reviews

**3** Not about transitions across care settings

**3** Review articles

**3** Descriptive studies or processes

**2** Abstracts

**2** No or unexplained interventions

**2** Technical articles without evaluation

**2** Newsletters or marketing material

**1** Brief summary of a survey

Literature Review Results (see main text for overview)

Ten articles about information transfer across care transitions were abstracted. We found three articles which described non-HIT interventions and seven articles which described HIT interventions.

Studies of non-HIT interventions to improve information transfer across care transitions

A study of post-discharge telephone calls for ten acute care hospitals assessed the number of attempted patient calls; the number of successful patient contacts; types of advice given to patients; and patient satisfaction[1]. Over 26,000 calls were made and about one-third resulted in patient contact. The types of advice given to patients were clarification of medication or home care instructions (34%), reminders for follow-up appointments or assistance with referrals (30%), return to the ED or their primary care physician (33%). There were 29 patients requiring immediate escalation of care. Patient satisfaction increased at one hospital with the mean score for “likelihood to recommend” increasing 2.5 points on a 100 point scale.

Another study of post-discharge telephone calls at one acute care hospital examined the number of successful patient contacts; the number of unmet clinical, appointment, or administrative needs; changes in the number of unmet needs by department over time; and patient satisfaction according to a survey[2]. They reported a 69% successful contact rate among 2,927 patients; in 14% of these contacts an unmet need was identified. The intervention resulted in a five point increase in patient satisfaction on a 100 point scale.

An observational study of a nurse-led intervention for children with complex care needs reported a significant decrease in emergency department visits from 470 to 398, a significant decrease in hospital admissions from 410 to 375, and a significant decrease in hospital bed days from 3,699 to 1,598 days[3]. Economic analysis indicated a cost savings of Australian $1.9 million per annum due to reduced ED visits and hospital bed-days.

Studies of HIT interventions for information transfer across care transitions

In addition to the three studies of non-HIT interventions, we found seven studies of HIT interventions. None of these studies described interoperable systems. A randomized controlled trial of a nurse practitioner-led (NP) care coordination intervention assessed whether patients had a PCP appointment within 21 days, readmission within 31 days, or ED visit within 31 days. The authors also assessed completion of hospital-recommended outpatient diagnostic work-ups[4]. They found a significant improvement in the number of patients who followed-up with their PCP within 21 days, as well as a significant improvement in a composite measure of all four outcomes.

Another study of an NP-led intervention for homebound elders was designed as an observational study[5]. The intervention included electronic documentation by the NP in the inpatient setting, electronic communication between the NP and the PCP while the patient was hospitalized, and electronic documentation by the NP about a post-discharge home visit in the outpatient record. The 30-day rehospitalization rate did not significantly decrease (16.6% to 15.8%) and mean hospital length of stay increased, but not significantly, from 6.15 days to 6.45. The annual cost to the program for the 1.6 FTE NPs was $197,000. Together, the two NPs generated $37,642 in billable services annually, representing 19% of their direct costs.

A randomized trial studied an HIT intervention that enabled discharging physicians to send information about diagnoses, medications, and pending test results to outpatient pharmacies and community-based providers [6]. The study examined readmission rates and found no significant difference between the intervention group and a control group. The authors discuss a better-than-average medication reconciliation process in the control group that may have nullified the results. They also point to a lack of closed-loop communication with receiving providers as an important contributor to readmissions.

Another study evaluated an electronic test result management tool that alerted physicians to results of tests that were pending at discharge. The authors tracked use of the electronic tool and surveyed discharging physicians about barriers to use [7]. Nearly half of discharging physicians did not use the tool. The survey revealed problems such as test results that were not clinically relevant and difficulties incorporating post-discharge results management into workflow.

A web-based tool for information transfer between SNFs and EDs was evaluated in a pre-post study [8]. The authors examined the rate of electronic referrals while concurrently surveying clinicians about adequacy of clinical information. The results showed that the tool was used in less than one-quarter of discharges on average with a wide variation in rate of electronic referrals. For example, at one nursing home the electronic referral rate dropped from 73% in the inception month of the study to 11% nine months later. The pre-post study design and the study setting limited the generalizability of the results.

We found one survey about two electronic documents that summarize patient-entered data, an emergency medical card and a continuity of care report [9]. Of note, these two electronic documents were created according to an early interoperability standard, called a Continuity of Care Record. Physicians were surveyed about whether the documents were useful for medical decision-making. About three-quarters of respondents thought that the documents were useful, particularly for decisions about medications, treatment decisions and ordering new labs. Though the information was not electronically transmitted in this study, the study serves as a proof of concept for interoperability standards.

Another study about discharge summaries used audit and surveys to assess different modes of transmitting discharge summaries to primary care providers, including email, fax, mail, and patient hand delivery[10]. The investigators called providers seven days post-discharge to determine the receipt rate and found that email and fax were more effective than mail or patient hand delivery. Providers preferred fax over mail, email, or patient hand delivery.

**Table 1: Results of Literature Review**

| **Authors** | **Title** | **Year** | **Study  Design** | **N** | **Population** | **Intervention** | **Outcomes** | **Results** |
| --- | --- | --- | --- | --- | --- | --- | --- | --- |
| Balaban RB,  Weissman JS, Samuel PA, Woolhandler S. | Redefining and redesigning hospital discharge to enhance patient care: a randomized controlled study | 2008 | RCT | 96 | Patients w/ PCP at 1 of 2 Massachusetts practices, admitted to Mass. hospital | 4-step: 1) user-friendly discharge form; 2) electronic transfer of discharge form to RNs at PCP practice; 3) phone contact by primary care RN to patient 4) PCP review and modification of discharge plan | 4 undesirable outcomes: 1) no outpatient appointment within 21 days; 2) 31-day readmission; 3) ED visits within 31 days; 4) failure to follow up on work-up recommended by inpatient team | Reduction in undesirable outcomes through intervention. 25.5% in intervention group, vs. 55.1% in control, and 55% in historical control |
| Chen Y, Brennan N, Magrabi F. | Is email an effective method for hospital discharge communication? | 2010 | RCT | 168 | Acute care hospital and 75 PCP practices of discharged patients, Sydney, Australia | Email discharge summary (control conditions: fax, mail, patient hand delivery) | % of discharge summaries received by PCP; survey of PCP practices | Fax and email were received at comparable rates (73.9% and 69.4%, respectively); both were significantly more effective than mail or patient hand delivery. 43 of 52 PCP (82.7%) practices responding to survey preferred fax. |
| Cochran VY,  Blair B, Wissinger L, Nuss TD. | Lessons Learned From Implementation of Postdischarge Telephone Calls at Baylor Health Care System | 2012 | Description of QI project | 26,803 | 10 Acute Care Hospitals, Texas | Post-discharge patient phone call | % of attempted patient calls, % of successful patient contacts; types of advice given to patients; patient satisfaction | 82%-100% attempted calls across hospitals, 20%-69% successful contacts; clarification of medication and/or home care instructions (n = 346, 34%), reminders for follow-up appointments or assistance with referrals (n = 314, 30%), return to the ED or their PCP (n = 346, 33%) |
| Dalal AK, Poon EG, Karson AS, Gandhi TK, Roy CL. | Lessons learned from implementation of a computerized application for pending tests at hospital discharge. | 2011 | Pilot intervention | 35 | 35 MDs at 2 Massachusetts hospitals | MDs tested using results manager to manage results of tests pending at discharge; survey of MDs | Frequency of use of results manager; barriers to use | 48% of MDs never used the application. Reasons include forgetting; seeing clinically irrelevant results; did not fit into workflow, too little time |
| Gombeski WR, Jr., Miller PJ, Hahn JH, Gillette CM, Belinson JL, Bravo LN, et al. | Patient callback program: a  quality improvement, customer service, and marketing tool. | 1993 | Description of QI project | 2927 | Patients discharged from overnight surgery stay at Ohio hospital | Follow-up phone call 21 days later, by trained interviewer using interview guide | % of patients reached, % with clinical needs | 69.3% reached. Of those reached, 14% had unmet clinical need. |
| Graumlich JF,  Novotny NL, Stephen Nace G, Kaushal H, Ibrahim-Ali W, Thei-vanayagam S, et al | Patient readmissions, emergency visits, and adverse events after software-assisted discharge from hospital: cluster randomized trial | 2009 | RCT | 70 MDs;  631  patients | Tertiary care hospital in Illinois; randomized at MD level | New software program enabling discharge provider to send outpatient pharmacist and community MD info on meds, pending tests, diagnoses | Readmission within 6 months | No difference at 6 months between intervention and control discharges in hospital readmissions, emergency department visits, or 1-month adverse events |
| Olola CHO,  Narus S, Nebeker J, Poynton M, Hales J, Rowan B, et al. | The perception of medical  professionals and medical students on the usefulness of an emergency medical card and a continuity of care report in enhancing continuity of care. | 2011 | Survey | 31 | Outpatient MDs and 4th-year med. students, Utah | 2 electronic documents to support clinical decision-making incorporating pt-entered data | Usefulness of the documents | 71% MDs and 78% students found the tools to be useful in influencing medical decision making at point of care, in particular: decisions about medications/ treatment decisions and ordering new labs. |
| Ornstein K, Smith KL, Foer DH, Lopez-Cantor MT, Soriano T. | To the hospital and back home again: A nurse practitioner-based transitional care program for hospitalized homebound people. | 2011 | Pre-post intervention study | 532 | Homebound elders (1,088 admissions over 27 month period), New York | NP sees patient in hospital; as post discharge home visit, NP writes inpatient progress note after admission to transmit outpatient information to the inpatient team, NP writes an electronic message to the outpatient team, NP writes a note in the outpatient record about the post discharge visit | Rehospitalization rate, length of stay, cost of the program | 30-day rehospitalization rate decreased from 16.6% to 15.8% (not significant). Mean LOS increased by 4.9%; no differences in mean LOS before/after for first time admits, 30-day admissions. Annual cost for the 1.6 FTE NPs = $197,000. The 2 NPs generated $37,642 in billable services annually, representing 19% of their direct costs. |
| Peter S, Chaney G, Zappia T, Van Veldhuisen C, Pereira S, Santamaria N. | Care coordination for children with complex care needs significantly reduces hospital utilization. | 2011 | Pre-post intervention study | 101 | Children with high utilization & complex needs at a Parth, West Australia, pediatric tertiary care hospital | Telephone support, detailed care plan, proactive outreach and management | ED utilization, hospital admissions, LOS | Significant decreases in ED (15%), hospital admissions (9%), and hospital bed days (43%). Cost savings of $A1.9 million /year |
| Zamora Z,  McCall B, Patel L, Biese K, Lamantia M, Platts-Mills T, et al. | Implementation of a web-based system to improve the transitional care of older adults. | 2012 | Retrospe-ctive intervention evaluation | 313 | ED and 4 surrounding SNFs in North Carolina | Web-based connection between hospital EHR and SNFs. 17 key elements could be entered by SNF in patient transfer, which would appear in EHR. SNF could also see ED info entered into system. | Compliance (using system); ED MDs satisfaction surveys | Compliance was 22.7% (i.e., using the system for referrals). Adequacy of info transferred improved based on ED MD surveys |

**ED – Emergency Department LOS – Length of Stay SNF – SNF NP – Nurse Practitioner PCP – Primary Care Physician RCT – Randomized Controlled Trial RN – Registered Nurse QI – Quality Improvement**

References

1. Cochran VY, Blair B, Wissinger L, Nuss TD. Lessons learned from implementation of postdischarge telephone calls at baylor health care system. J Nurs Adm. 2012 /;42(1):40-6.

2. Gombeski WR,Jr, Miller PJ, Hahn JH, Gillette CM, Belinson JL, Bravo LN, Curry PS. Patient callback program: A quality improvement, customer service, and marketing tool. J Health Care Mark. 1993 Fall;13(3):60-5.

3. Peter S, Chaney G, Zappia T, Van Veldhuisen C, Pereira S, Santamaria N. Care coordination for children with complex care needs significantly reduces hospital utilization. J SPEC PEDIATR NURS. 2011 10;16(4):305-12.

4. Balaban RB, Weissman JS, Samuel PA, Woolhandler S. Redefining and redesigning hospital discharge to enhance patient care: A randomized controlled study. J Gen Intern Med. 2008 Aug;23(8):1228-33.

5. Ornstein K, Smith KL, Foer DH, Lopez-Cantor MT, Soriano T. To the hospital and back home again: A nurse practitioner-based transitional care program for hospitalized homebound people. J Am Geriatr Soc. 2011 Mar;59(3):544-51.

6. Graumlich JF, Novotny NL, Nace GS, Aldag JC. Patient and physician perceptions after software-assisted hospital discharge: Cluster randomized trial. J Hosp Med. 2009 /;4(6):356-63.

7. Dalal AK, Poon EG, Karson AS, Gandhi TK, Roy CL. Lessons learned from implementation of a computerized application for pending tests at hospital discharge. J Hosp Med. 2011 /;6(1):16-21.

8. Zamora Z, McCall B, Patel L, Biese K, Lamantia M, Platts-Mills T, Naus N, Jerkewitz H, P., Cairns C, B., Busby-Whitehead J, Kizer J, S. Implementation of a web-based system to improve the transitional care of older adults. J Nurs Care Qual. 2012 2012;27(2):182-9.

9. Olola CHO, Narus S, Nebeker J, Poynton M, Hales J, Rowan B, LeSieur H, Zumbrennen C, Edwards AA, Crawford R, Amundsen S, Kabir Y, Atkin J, Newberry C, Young J, Hanifi T, Risenmay B, Sorensen T, Evans RS. The perception of medical professionals and medical students on the usefulness of an emergency medical card and a continuity of care report in enhancing continuity of care. Int J Med Informatics. 2011 /;80(6):412-20.

10. Chen Y, Brennan N, Magrabi F. Is email an effective method for hospital discharge communication? A randomized controlled trial to examine delivery of computer-generated discharge summaries by email, fax, post and patient hand delivery. Int J Med Inform. 2010 Mar;79(3):167-72.

**Search strategy and consensus review**

Pubmed May 6, 2012

((("Patient Transfer"[MeSH Terms] OR "Patient Discharge"[MAJR] OR "care transition"[tiab] OR "care transitions"[tiab]) AND "Continuity of Patient Care/organization and administration"[MAJR])) AND (((((("Computing Methodologies"[Mesh]) OR "Information Dissemination"[Mesh]) OR "Information Services"[Mesh]) OR "Telecommunications"[Mesh]) OR "Medical Informatics"[Mesh]) OR "Medical Records Systems, Computerized"[Mesh])

CINAHL May 7, 2012

(((MH "Information Science+")) AND (S11 and S12)) AND AB (transition OR transitions OR continuity )

Embase May 15, 2012

No. Query Results

#9 #4 AND #8 **3**

#8 #2 AND #7 **90**

#7 'hospital'/exp AND 'nursing home'/exp **5,547**

#6 'clinician centered approach' OR 'medical information system' OR 'patient care' **486,606**

#5 #3 AND #4 **266**

#4 'information system'/exp **97,605**

#3 #1 AND #2 **6,253**

#2 transition OR continuity OR handoff **223,933**

#1 'patient care'/exp OR 'patient care planning'/exp **447,171**

Title list

1. Continuity of care scoring guidelines (intents and score 1s) for the 1991 CSM ("Patient Management" chapter). Joint Commission Perspectives. 1991 Jan-Feb;11(1):suppl I1-2.
2. Automated discharge summary enhances communication. Hospital Peer Review. 1997 Jan;22(1):7-8.
3. Discharge process enables faster placement. Hospital Case Management. 2002 Apr;10(4):59-61.
4. Discharge Planning Advisor--the update for improving continuity of care. Successful CM program gets JCAHO approval. Hospital Peer Review. 2004 Apr;29(4):51-4.
5. Discharge initiatives help transition patients through continuum of care. Hospital Case Management. 2004 Apr;12(4):49-53.
6. Technology combo yields high satisfaction rates: using systems in combination was essential. ED Management. 2005;17(10):116-8.
7. Main barriers to effective handoffs identified. Healthcare Benchmarks Qual Improv. 2006 Feb;13(2):17-9.
8. Using medication reconciliation to prevent errors. Sentinel Event Alert. 2006 Jan 23(35):1-4.
9. Technology. Direct line for discharge notes. Health Service Journal. 2010 Feb 25;120(6195):26.
10. Study finds ways to boost care coordination between emergency and primary care providers. ED Management. 2011;23(6):65-7.
11. Hospitals, providers collaborate on transitions. Hospital Case Management. 2012 Jan;20(1):11-2.
12. Committee opinion No. 517: Communication strategies for patient handoffs. Obstetrics and gynecology. 2012;119(2 PART 1):408-11.
13. Abad-Corpa E, Carrillo-Alcaraz A, Royo-Morales T, Perez-Garcia MC, Rodriguez-Mondejar JJ, Saez-Soto A, et al. Effectiveness of planning hospital discharge and follow-up in primary care for patients with chronic obstructive pulmonary disease: research protocol. Journal of Advanced Nursing. 2010 Jun;66(6):1365-70.
14. Abraham J, Kannampallil TG, Patel VL. Bridging gaps in handoffs: A continuity of care based approach. Journal of Biomedical Informatics. 2012;45(2):240-54.
15. Abraham J, Reddy MC. Challenges to inter-departmental coordination of patient transfers: a workflow perspective. Int J Med Inform. 2010 Feb;79(2):112-22.
16. Anderson MA, Helms L. Home health care referrals following hospital discharge: communication in health services delivery. Hospital and Health Services Administration. 1993 Winter;38(4):537-55.
17. Appleby C. NYCLIX: New York HIE Life: Aan expansive HIE network has taken shape in the nation's most densely populated urban area. Healthcare Informatics. 2010;27(10):29-31.
18. Arbaje AI, Maron DD, Yu Q, Wendel VI, Tanner E, Boult C, et al. The Geriatric Floating Interdisciplinary Transition Team. Journal of the American Geriatrics Society. 2010;58(2):364-70.
19. Arora VM. Tackling care transitions: Mom and apple pie vs. the devil in the details. Journal of General Internal Medicine. 2009 Aug;24(8):985-7.
20. Balaban RB, Weissman JS, Samuel PA, Woolhandler S. Redefining and redesigning hospital discharge to enhance patient care: a randomized controlled study. Journal of General Internal Medicine. 2008 Aug;23(8):1228-33.
21. Barr P. Cutting readmissions: Index allows hospital to track high-risk patients. Modern Healthcare. 2011(Journal Article):10-.
22. Bell CM, Brener SS, Comrie R, Anderson GM, Bronskill SE. Quality measures for medication continuity in long-term care facilities, using a structured panel process. Drugs and Aging. 2012;29(4):319-27.
23. Berta W, Barnsley J, Bloom J, Cockerill R, Davis D, Jaakkimainen L, et al. Enhancing continuity of information: essential components of a referral document. Canadian Family Physician. 2008;54(10):1432.
24. Blobel B. Interoperable healthcare information system components for continuity of care. British Journal of Healthcare Computing & Information Management. 2003;20(7):22-4.
25. Bolton P. A review of the role of information technology in discharge communications in Australia. Australian Health Review. 1999;22(3):56-64.
26. Boockvar KS, Liu S, Goldstein N, Nebeker J, Siu A, Fried T. Prescribing discrepancies likely to cause adverse drug events after patient transfer. Quality and Safety in Health Care. 2009;18(1):32-6.
27. Burstein HJ. The continuity of care checklist: A critical frontier in patient safety. JNCCN Journal of the National Comprehensive Cancer Network. 2011;9(11):1217-8.
28. Buysse H, Coorevits P, Thienpont G, De Moor G. Trans-eCare: creating a transparent data exchange platform. Studies in Health Technology and Informatics. 2008;141(Journal Article):38-44.
29. Cacciola JS, Camilleri AC, Carise D, Rikoon SH, McKay JR, McLellan AT, et al. Extending residential care through telephone counseling: Initial results from the Betty Ford Center Focused Continuing Care protocol. Addictive Behaviors. 2008;33(9):1208-16.
30. Callahan ST, Winitzer RF, Keenan P. Transition from pediatric to adult-oriented health care: a challenge for patients with chronic disease. Current Opinion in Pediatrics. 2001 Aug;13(4):310-6.
31. Calleja P, Aitken LM, Cooke ML. Information transfer for multi-trauma patients on discharge from the emergency department: mixed-method narrative review. Journal of Advanced Nursing. 2011 Jan;67(1):4-18.
32. Carlisle D. Electronic discharge: Don't leave empty handed. Health Service Journal. 2011 Sep 22;121(6275):24-5.
33. Chen Y, Brennan N, Magrabi F. Is email an effective method for hospital discharge communication? A randomized controlled trial to examine delivery of computer-generated discharge summaries by email, fax, post and patient hand delivery. International journal of medical informatics. 2010;79(3):167-72.
34. Cibulskis CC, Giardino AP, Moyer VA. Care transitions from inpatient to outpatient settings: ongoing challenges and emerging best practices. Hosp Pract (Minneap). 2011 Aug;39(3):128-39.
35. Cochran VY, Blair B, Wissinger L, Nuss TD. Lessons Learned From Implementation of Postdischarge Telephone Calls at Baylor Health Care System. Journal of Nursing Administration. 2012;42(1):40-6.
36. Collins SA, Bakken S, Vawdrey DK, Coiera E, Currie LM. Agreement between common goals discussed and documented in the ICU. Journal of the American Medical Informatics Association. 2011;18(1):45-50.
37. Collins SA, Stein DM, Vawdrey DK, Stetson PD, Bakken S. Content overlap in nurse and physician handoff artifacts and the potential role of electronic health records: A systematic review. Journal of Biomedical Informatics. 2011;44(4):704-12.
38. Crane AB. The payoff: preventing errors. Medication management. Hospitals and Health Networks. 2007 Oct;81(10):57-8, 60, 2.
39. Dalal AK, Poon EG, Karson AS, Gandhi TK, Roy CL. Lessons learned from implementation of a computerized application for pending tests at hospital discharge. Journal of Hospital Medicine. 2011;6(1):16-21.
40. Davis JL, Saunders TA, Terrazas E, Rabban JT. A web-based tracking system to facilitate transfer of patient care between residents in a multi-site academic anatomic pathology department: A solution to JCAHO and acgme mandates for optimizing patient nullHandoffsnull. Laboratory Investigation. 2012;92(Journal Article):498A.
41. Dickerson AE, Sensmeier J. Sharing data to ensure continuity of care. Nurs Manage. 2010 Jul;41(7):19-22.
42. Ferranti JM, Musser RC, Kawamoto K, Hammond WE. The clinical document architecture and the continuity of care record: a critical analysis. Journal of the American Medical Informatics Association. 2006;13(3):245-52.
43. Foust JB, Vuckovic N, Henriquez E. Hospital to Home Health Care Transition: Patient, Caregiver, and Clinician Perspectives. Western journal of nursing research. 2012;34(2):194-212.
44. Freitag M, Carroll VS. Handoff communication: using failure modes and effects analysis to improve the transition in care process. Quality management in health care. 2011;20(2):103-9.
45. Frisse ME. Health information exchange in Memphis: impact on the physician-patient relationship. Journal of Law, Medicine & Ethics. 2010;38(1):50-7.
46. Gandara E, Ungar J, Lee J, Chan-Macrae M, O'Malley T, Schnipper JL. Discharge documentation of patients discharged to subacute facilities: a three-year quality improvement process across an integrated health care system. Joint Commission journal on quality and patient safety / Joint Commission Resources. 2010 Jun;36(6):243-51.
47. Gardner RL. Successful interventions for avoiding readmission in the elderly. Medicine and Health, Rhode Island. 2008 Sep;91(9):285-7.
48. Glintborg B, Andersen SE, Dalhoff K. Insufficient communication about medication use at the interface between hospital and primary care. Quality & Safety in Health Care. 2007;16(1):34-9.
49. Gombeski WR, Jr., Miller PJ, Hahn JH, Gillette CM, Belinson JL, Bravo LN, et al. Patient callback program: a quality improvement, customer service, and marketing tool. Journal of Health Care Marketing. 1993 Fall;13(3):60-5.
50. Graumlich JF, Novotny NL, Nace GS, Aldag JC. Patient and physician perceptions after software-assisted hospital discharge: Cluster randomized trial. Journal of Hospital Medicine. 2009;4(6):356-63.
51. Graumlich JF, Novotny NL, Stephen Nace G, Kaushal H, Ibrahim-Ali W, Theivanayagam S, et al. Patient readmissions, emergency visits, and adverse events after software-assisted discharge from hospital: cluster randomized trial. Journal of hospital medicine : an official publication of the Society of Hospital Medicine. 2009 Sep;4(7):E11-9.
52. Gruneir A, Dhalla IA, van Walraven C, Fischer HD, Camacho X, Rochon PA, et al. Unplanned readmissions after hospital discharge among patients identified as being at high risk for readmission using a validated predictive algorithm. Open Med. 2011;5(2):e104-11.
53. Hagglund M, Chen R, Koch S. Modeling shared care plans using CONTsys and openEHR to support shared homecare of the elderly. Journal of the American Medical Informatics Association. 2011;18(1):66-9.
54. Halamka J, Aranow M, Ascenzo C, Bates D, Debor G, Glaser J, et al. Health care IT collaboration in Massachusetts: the experience of creating regional connectivity. Journal of the American Medical Informatics Association. 2005;12(6):596-601.
55. Harte B, Hixson E, Proctor A, LaRochelle D, Auron M. Actionable quality improvement: Synergizing the EMR and BI. Journal of Hospital Medicine. 2012;7(Journal Article):S131-S2.
56. Helleso R. Information handling in the nursing discharge note. Journal of Clinical Nursing. 2006 Jan;15(1):11-21.
57. Holen KA. Post-hospital transition to a skilled nursing facility--Compliance, competence, and communication. Journal of Gerontological Nursing. 2006 Sep;32(9):5-9.
58. Holland DE, Hemann MA. Standardizing hospital discharge planning at the Mayo Clinic. Joint Commission journal on quality and patient safety / Joint Commission Resources. 2011 Jan;37(1):29-36.
59. Hoppszallern S. Taking the Guesswork Out of Transitions in Patient Care. H&HN: Hospitals & Health Networks. 2011;85(6):40-.
60. Horner K, Wagner E, Tufano J. Electronic consultations between primary and specialty care clinicians: early insights. Issue brief (Commonwealth Fund). 2011;23(Journal Article):1-14.
61. Hubner U, Flemming D, Heitmann KU, Oemig F, Thun S, Dickerson A, et al. The need for standardised documents in continuity of care: results of standardising the eNursing summary. Studies in Health Technology and Informatics. 2010;160(Pt 2):1169-73.
62. [REPEAT OF 61] Hubner U, Flemming D, Heitmann KU, Oemig F, Thun S, Dickerson A, et al. The need for standardised documents in continuity of care: results of standardising the eNursing summary. Studies in Health Technology and Informatics. 2010;160(Pt 2):1169-73.
63. Hunter J, Freer Y, Gatt A, Reiter E, Sripada S, Sykes C, et al. BT-Nurse: computer generation of natural language shift summaries from complex heterogeneous medical data. Journal of the American Medical Informatics Association. 2011;18(5):621-4.
64. Jefford MJ, Clancy JL, Butler SM. A quality use of medicines program for continuity of care in therapeutics from hospital to community. Medical Journal of Australia. 2002 Nov 18;177(10):574-5; author reply 5-6.
65. Kalanithi L, Coffe C, Green A, Ranji S, Vidyarthi A, Baron R. A novel financial incentive program for hospital-based residents to improve communication with inpatients primary care physicians. Journal of General Internal Medicine. 2010;25(Journal Article):S459.
66. Kannry J, Moore C. MediSign: using a web-based SignOut System to improve provider identification. Proceedings / Amia Symposium. 1999:550-4.
67. Karapinar F, van den Bemt PM, Zoer J, Nijpels G, Borgsteede SD. Informational needs of general practitioners regarding discharge medication: content, timing and pharmacotherapeutic advice. Pharmacy World and Science. 2010 Apr;32(2):172-8.
68. Kejriwal K, Israel C, Wilde G. Transitions of frail seniors across the spectrum of healthcare. Journal of the American Medical Directors Association. 2010;11(3):B26.
69. Kirkley D, Brown D, Lester V. Information systems & technology. Clinical IT: building a bridge between acute and ambulatory care. Nursing economic$. 2004;22(1):36.
70. Kleifgen B, Combs D, Walker G, Franke H, Cramton R. Use of rapid PDSA cycles to improve pediatric resident handoffs. Journal of Hospital Medicine. 2012;7(Journal Article):S98.
71. Knaup P, Pilz J, Kaltschmidt J, Ludt S, Szecsenyi J, Haefeli WE. Standardized documentation of drug recommendations in discharge letters--a contribution to quality management in cooperative care. Methods of Information in Medicine. 2006;45(4):336-42.
72. Konetzka RT, Werner RM. Applying market-based reforms to long-term care. Health affairs. 2010;29(1):74-80.
73. Kramer JS, Hopkins PJ, Rosendale JC, Garrelts JC, Hale LS, Nester TM, et al. Implementation of an electronic system for medication reconciliation. American Journal of Health-System Pharmacy. 2007 Feb 15;64(4):404-22.
74. LaMantia MA, Scheunemann LP, Viera AJ, Busby-Whitehead J, Hanson LC. Interventions to improve transitional care between nursing homes and hospitals: a systematic review. Journal of the American Geriatrics Society. 2010;58(4):777-82.
75. Laxmisan A, Hakimzada F, Sayan OR, Green RA, Zhang J, Patel VL. The multitasking clinician: decision-making and cognitive demand during and after team handoffs in emergency care. Int J Med Inform. 2007 Nov-Dec;76(11-12):801-11.
76. Lenhard RE, Jr., Buchman JP, Achuff SC, Kahane SN, Macmanus CJ. AUTRES--the Johns Hopkins Hospital automated resume. Journal of Medical Systems. 1991 Jun;15(3):237-47.
77. Lin JJ, Moore C. Impact of an Electronic Health Record on Follow-up Time for Markedly Elevated Serum Potassium Results. American Journal of Medical Quality. 2011;26(4):308-14.
78. Lindsay D. Computer connection. Nursing BC. 1995 Mar-Apr;27(2):16-7.
79. Lyhne S, Georgiou A, Marks A, Tariq A, Westbrook JI. Towards an understanding of the information dynamics of the handover process in aged care settings-A prerequisite for the safe and effective use of ICT. International journal of medical informatics. 2012(Journal Article).
80. Magilvy JK, Lakomy JM. Transitions of older adults to home care. Home Health Care Services Quarterly. 1991;12(4):59-70.
81. Maher RL, Hajjar ER. Medication errors in the ambulatory elderly. Aging Health. 2012;8(2):127-35.
82. Manning S, Wendler MC, Baur K. An innovative approach to standardizing heart failure care: the heart failure support team. Journal of the American Academy of Nurse Practitioners. 2010;22(8):417-23.
83. Manser T, Foster S. Effective handover communication: An overview of research and improvement efforts. Best Practice and Research: Clinical Anaesthesiology. 2011;25(2):181-91.
84. Mantone J. Bridging the gap. Network to link hospitals, skilled-nursing facilities. Modern Healthcare. 2003 Jun 23;33(25):46.
85. Martin JR, Aubeneau C, Geniteau E. [The synthesis of care]. Revue de l Infirmiere. 2009 May(150):29-31.
86. Mathur R, Clark RA, Dhillon DP, Winter JH, Lipworth BJ. A repeat audit of hospital discharge letters in patients admitted with acute asthma. Scottish Medical Journal. 1997 Feb;42(1):19-21.
87. Mikos K. Monitoring handoffs for standardization. Nurs Manage. 2007 Dec;38(12):16-20.
88. Miller D, Schapira M, Visotcky A, Laud P, Arora V, Fletcher K. Hospital handoffs: A descriptive analysis of written sign-out content and an exploration of a sign-out quality assessment tool. Journal of General Internal Medicine. 2011;26(Journal Article):S318-S9.
89. Mishra AK, Bhattarai S, Bhurtel P, Bista NR, Shrestha P, Thakali K, et al. Need for improvement of medical records. JNMA; journal of the Nepal Medical Association. 2009 Apr-Jun;48(174):103-6.
90. Mistry NK, Toulany A, Edmonds JF, Matlow A. Optimizing physician handover through the creation of a comprehensive minimum data set. Healthcare Quarterly. 2010;13(Journal Article):102-9.
91. Moore P, Armitage G, Wright J, Dobrzanski S, Ansari N, Hammond I, et al. Medicines reconciliation using a shared electronic health care record. J Patient Saf. 2011 Sep;7(3):148-54.
92. Nabors C, Peterson SJ, Lee WN, Mumtaz A, Shah T, Sule S, et al. Experience with faculty supervision of an electronic resident sign-out system. American Journal of Medicine. 2010 Apr;123(4):376-81.
93. Nabors C, Peterson SJ, Sule S, Forman L, Kerpen H, Schwarcz MD, et al. Tracking outpatient continuity and chronic disease indicators-A novel use of the new innovations clinic module. American Journal of Therapeutics. 2012;19(2):76-80.
94. Nelson EC, Batalden PB, Huber TP, Mohr JJ, Godfrey MM, Headrick LA, et al. Microsystems in health care: part 1. Learning from high-performing front-line clinical units. Joint Commission Journal on Quality Improvement. 2002;28(9):472-93.
95. Nelson JM, Carrington JM. Transitioning the older adult in the ambulatory care setting. AORN Journal. 2011;94(4):348-61.
96. New PW. A quality use of medicines program for continuity of care in therapeutics from hospital to community. Medical Journal of Australia. 2002 Nov 18;177(10):575; author reply -6.
97. Nichols DG. Future of Welch Library. Baltimore MD: Johns Hopkins University; 2011. p. 1.
98. Nosbusch JM, Weiss ME, Bobay KL. An integrated review of the literature on challenges confronting the acute care staff nurse in discharge planning. Journal of Clinical Nursing. 2011;20(5):754-74.
99. Olola CHO, Narus S, Nebeker J, Poynton M, Hales J, Rowan B, et al. The perception of medical professionals and medical students on the usefulness of an emergency medical card and a continuity of care report in enhancing continuity of care. International journal of medical informatics. 2011;80(6):412-20.
100. Olvera L, Bliss MC. Perfecting the patient handoff: improving the process for patients and nurses. Nurs Womens Health. 2010 Dec-2011 Jan;14(6):496-504.
101. Ong MS, Coiera E. A systematic review of failures in handoff communication during intrahospital transfers. Joint Commission journal on quality and patient safety / Joint Commission Resources. 2011 Jun;37(6):274-84.
102. Ornstein K, Smith KL, Foer DH, Lopez-Cantor MT, Soriano T. To the hospital and back home again: A nurse practitioner-based transitional care program for hospitalized homebound people. Journal of the American Geriatrics Society. 2011;59(3):544-51.
103. Palma JP, Sharek PJ, Longhurst CA. Impact of electronic medical record integration of a handoff tool on sign-out in a newborn intensive care unit. Journal of Perinatology. 2011;31(Journal Article):S83-4.
104. Palmiere G. Security in the wake of change. Health Management Technology. 2002 Jan;23(1):26, 8, 31.
105. Parrish MM, O'Malley K, Adams RI, Adams SR, Coleman EA. Implementation of the Care Transitions Intervention: sustainability and lessons learned. Professional case management. 2009;14(6):282-95.
106. Paterson JM, Allega RL. Improving communication between hospital and community physicians. Feasibility study of a handwritten, faxed hospital discharge summary. Discharge Summary Study Group. Canadian Family Physician. 1999 Dec;45:2893-9.
107. Patterson PK, Blehm R, Foster J, Fuglee K, Moore J. Nurse information needs for efficient care continuity across patient units. Journal of Nursing Administration. 1995 Oct;25(10):28-36.
108. Pesanka DA, Greenhouse PK, Rack LL, Delucia GA, Perret RW, Scholle CC, et al. Ticket to ride: reducing handoff risk during hospital patient transport. Journal of Nursing Care Quality. 2009 Apr-Jun;24(2):109-15.
109. Peter S, Chaney G, Zappia T, Van Veldhuisen C, Pereira S, Santamaria N. Care coordination for children with complex care needs significantly reduces hospital utilization. Journal for Specialists in Pediatric Nursing. 2011;16(4):305-12.
110. Petersen LA, Orav EJ, Teich JM, O'Neil AC, Brennan TA. Using a computerized sign-out program to improve continuity of inpatient care and prevent adverse events. Joint Commission Journal on Quality Improvement. 1998;24(2):77-87.
111. Phillips A. The effect of a standardized form in guiding communication between peers during the hand-off of patients in a hospital setting. Studies in Health Technology and Informatics. 2009;146:885.
112. Politi MC, Pieterse AH, Truant T, Borkhoff C, Jha V, Kuhl L, et al. Interprofessional education about patient decision support in specialty care. Journal of interprofessional care. 2011;25(6):416-22.
113. Pollard C, Bailey KA, Petitte T, Baus A, Swim M, Hendryx M. Electronic patient registries improve diabetes care and clinical outcomes in rural community health centers. Journal of Rural Health. 2009;25(1):77-84.
114. Priest A. Bridging the gap between acute care and community care. Nursing BC. 2006 Oct;38(4):16-9.
115. Rafter RH, Kelly TM. Nursing implementation of a telestroke programme in a community hospital in the US. Journal of nursing management. 2011;19(2):193-200.
116. Ray LN. "A Ticket to Ride" protects patients off the unit. Nursing. 2009 May;39(5):57-8.
117. Reiner BI. Medical imaging data reconciliation, part 2: Clinical order entry and imaging report data reconciliation. JACR Journal of the American College of Radiology. 2011;8(10):720-4.
118. Reiner BI. Medical imaging data reconciliation, part 1: Innovation opportunity. JACR Journal of the American College of Radiology. 2011;8(9):622-5.
119. Reti SR, Feldman HJ, Ross SE, Safran C. Improving personal health records for patientcentered care. Journal of the American Medical Informatics Association. 2010;17(2):192-5.
120. Rimaz R, Waldmann F, Bula C. [Between home and institution: network meetings as a decision aide]. Revue Medicale de la Suisse Romande. 2000 Nov;120(11):893-6.
121. Rudiger-Sturchler M, Keller DI, Bingisser R. Emergency physician intershift handover - can a dINAMO checklist speed it up and improve quality? Swiss Med Wkly. 2010;140:w13085.
122. Rudin RS, Salzberg CA, Szolovits P, Volk LA, Simon SR, Bates DW. Care transitions as opportunities for clinicians to use data exchange services: how often do they occur? Journal of the American Medical Informatics Association. 2011;18(6):853-8.
123. Sachdeva AK. Invited commentary: Use of information technology to support safe and efficient transfer of care in surgery. Surgery. 2004 Jul;136(1):14-5.
124. Saufl NM. Reconciliation of medications. Journal of Perianesthesia Nursing. 2006 Apr;21(2):126-7.
125. Scavuzzo J, Gamba N. Bridging the gap: the virtual chemotherapy unit. Journal of Pediatric Oncology Nursing. 2004;21(1):27-32.
126. Schnall R, Gordon P, Camhi E, Bakken S. Perceptions of factors influencing use of an electronic record for case management of persons living with HIV. AIDS Care. 2011;23(3):357-65.
127. Shaaban R. Standardization of discharge documentation to post acute care (PAC) facilities R Shaaban, do, P Owusu-Griffin, MD baystate medical center. Journal of Hospital Medicine. 2012;7(Journal Article):S95.
128. Shah M, Alvarez K, Berge C, Johnson V, Reed G. Cost avoidance through the use of a dedicated clinical pharmacy specialist on a hospitalist service. Journal of Hospital Medicine. 2012;7(Journal Article):S60.
129. Shah P, Boudos R. Transitions from adolescent to adult care. Pediatric annals. 2012;41(2):73-8.
130. Siegler EL, Murtaugh CM, Rosati RJ, Clark A, Ruchlin H, Sobolewski S, et al. Improving the transition to home healthcare by rethinking the purpose and structure of the CMS 485: first steps. Home Health Care Services Quarterly. 2006;25(3-4):27-38.
131. Siemsen IM, Michaelsen L, Nielsen J, Ostergaard D, Andersen HB. [Patient handover involves numerous safety risks]. Ugeskrift for Laeger. 2011 May 16;173(20):1412-6.
132. Singh H, Thomas EJ, Mani S, Sittig D, Arora H, Espadas D, et al. Timely follow-up of abnormal diagnostic imaging test results in an outpatient setting: are electronic medical records achieving their potential? Archives of Internal Medicine. 2009;169(17):1578-86.
133. Soong K, Zlokas DA, Tyagi S, Hodes DZ, Gennari A, Ruby CM. Impact of a pharmacist as a member of an interprofessional team to identify and reduce medication related problems during transitions of care from skilled nursing facilities (SNFs) to home. Pharmacotherapy. 2011;31(10):426e.
134. Sparbel KJH, Anderson MA. Clinical scholarship. Integrated literature review of continuity of care: part 1, conceptual issues. Journal of Nursing Scholarship. 2000;32(1):17-24.
135. Staggers N, Clark L, Blaz JW, Kapsandoy S. Why patient summaries in electronic health records do not provide the cognitive support necessary for nurses' handoffs on medical and surgical units: insights from interviews and observations. Health informatics journal. 2011;17(3):209-23.
136. Staggers N, Jennings BM. The content and context of change of shift report on medical and surgical units. Journal of Nursing Administration. 2009;39(9):393-8.
137. Stainkey L, Pain T, McNichol M, Hack J, Roberts L. Matched comparison of GP and consultant rating of electronic discharge summaries. HIM J. 2010;39(3):7-15.
138. Stein DM, Stetson PD. Commentary: Time to sign off on signout. Academic Medicine. 2011;86(7):804-6.
139. Stoicu-Tivadar L, Stoicu-Tivadar V, Berian D. Tele-assistance for discharged patients supporting continuity of care. Studies in Health Technology and Informatics. 2010;155:170-5.
140. Sullivan F, Wilson P. The completeness and accuracy of patient record transfer between practices. Health Bulletin. 1997 Jan;55(1):16-9.
141. Terrell KM, Miller DK. Strategies to Improve Care Transitions Between Nursing Homes and Emergency Departments. Journal of the American Medical Directors Association. 2011;12(8):602-5.
142. Tess A, O'Neill S, Yang J. Discharge appointment service: Supporting house staff and improving transitions of care. Journal of Hospital Medicine. 2011;6(4):S138-S9.
143. Thomas PL. Case manager role definitions: do they make an organizational impact? Professional case management. 2008 Mar-Apr;13(2):61-71; quiz 2-3.
144. Thompson R, Ferris T. A novel approach to measuring continuity of hospital care. Journal of General Internal Medicine. 2011;26(Journal Article):S219-S20.
145. Tripp JS, Narus SP, Magill MK, Huff SM. Evaluating the accuracy of existing EMR data as predictors of follow-up providers. Journal of the American Medical Informatics Association. 2008;15(6):787-90.
146. Tsilimingras D, Brummel-Smith K, Brooks RG. Postdischarge adverse events in the elderly. J Patient Saf. 2009 Dec;5(4):201-4.
147. Van Eaton EG, Horvath KD, Lober WB, Pellegrini CA. Organizing the transfer of patient care information: the development of a computerized resident sign-out system. Surgery. 2004 Jul;136(1):5-13.
148. Van Walraven C, Taljaard M, Bell CM, Etchells E, Stiell IG, Zarnke K, et al. A prospective cohort study found that provider and information continuity was low after patient discharge from hospital. Journal of clinical epidemiology. 2010;63(9):1000-10.
149. Vinker S, Kitai E, Or Y, Nakar S. Primary care follow up of patients discharged from the emergency department: a retrospective study. BMC Fam Pract. 2004 Aug 7;5:16.
150. Wayne JD, Tyagi R, Reinhardt G, Rooney D, Makoul G, Chopra S, et al. Simple standardized patient handoff system that increases accuracy and completeness. J Surg Educ. 2008 Nov-Dec;65(6):476-85.
151. Weiner M, Callahan CM, Tierney WM, Overhage JM, Mamlin B, Dexter PR, et al. Using information technology to improve the health care of older adults. Annals of Internal Medicine. 2003;139(5):430-6.
152. Weitzman ER, Kaci L, Quinn M, Mandl KD. Helping high-risk youth move through high-risk periods: personally controlled health records for improving social and health care transitions. Journal of diabetes science and technology. 2011 Jan;5(1):47-54.
153. Welsh C, Ludwig-Beymer P. Shortened lengths of stay: ensuring continuity of care for mothers and babies. Lippincotts Primary Care Practice. 1998 May-Jun;2(3):284-91.
154. Were MC, Li X, Kesterson J, Cadwallader J, Asirwa C, Khan B, et al. Adequacy of hospital discharge summaries in documenting tests with pending results and outpatient follow-up providers. Journal of General Internal Medicine. 2009 Sep;24(9):1002-6.
155. White JC, Sarti J. The transfer of patients' ethics information among cooperating institutions: a future function of ethics networks. HEC Forum. 1993 Nov;5(6):362-7.
156. Willis J, White R, Thirlwell S, Santiago M, Ernst A, Jepsen A, et al. Outpatient BMT electronic nursing documentation: One stop charting. Biology of Blood and Marrow Transplantation. 2012;18(2):S388.
157. Wilson R. Improving clinical handover in emergency departments. Emergency Nurse. 2011 Apr;19(1):22-6.
158. Ye K, Taylor DM, Knott JC, Dent A, MacBean CE. Handover in the emergency department: deficiencies and adverse effects. Emergency Medicine Australasia. 2007;19(5):433-41.
159. Young B. Medication reconciliation matters. Medsurg Nursing. 2008 Oct;17(5):332-6.
160. Zamora Z, McCall B, Patel L, Biese K, Lamantia M, Platts-Mills T, et al. Implementation of a web-based system to improve the transitional care of older adults. Journal of Nursing Care Quality. 2012;27(2):182-9.

SECOND ROUND CITATIONS (6/25/12):

1. System uses technology to deliver higher quality, safety. Modern Healthcare. 2008;38(28):35-.
2. Preferred Practice 3. Preferred Practices and Performance Measures for Measuring and Reporting Care Coordination: A Concensus Report from the National Quality Forum. Washington DC: NQF2010 Contract No.: Report.
3. Boost care coordination between ED, primary care. Healthcare Benchmarks & Quality Improvement. 2011;18(8):94-6.
4. Army Health Care: Progress Made in Staffing and Monitoring Units that Provide Outpatient Case Management, but Additional Steps Needed: General Accounting Office Contract No.: Report.
5. Community Based Outpatient Clinic Reviews Bradford (McKean County) and Franklin (Venango County), PA Camp Hill and Pottsville/Frackville, PA Mission, SD and Newcastle, WY Hibbing and Rochester, MN Contract No.: Report.
6. Health Information Exchange in Post-Acute and Long-Term Care: Office of the Assistant Secretary for Planning and Evaluation Contract No.: Report.
7. Transition of Care for Acute Stroke and Myocardial Infarction Patients: From Hospitalization to Rehabilitation, Recovery, and Secondary Prevention. Executive Summary. Evidence Report/Technology Assessment Number 202: National Center for Health Services Research Contract No.: Report.
8. HAA update: hospice forum. Caring. 2003;22(9):42-.
9. Longitudinal records enable instant QI changes. Healthcare Benchmarks & Quality Improvement. 2004;11(12):136-8.
10. My scope of practice. Making house calls wherever the patient calls home... Scott Bolhack, MD, CMD, FACP. Ostomy Wound Management. 2004;50(10):16.
11. Winning the battle for standardization. Health Management Technology. 2007;28(10):34.
12. Afilalo M. The Impact of a Standardized Information System between the Emergency Department and the Primary Care Network: Effects on Continuity and Quality of Care. Montreal Quebec: Canadian Health Research Foundation2003 Contract No.: Report.
13. Anderson MA. Talking about patients: communication and continuity of care. 2000;14(Journal Article):15-28.
14. Anderson MA, Foreman MD, Theis SL, Helms LB. Issues in clinical nursing research - Unanticipated results of continuity of care research with the elderly .1. Design issues. Western journal of nursing research. 1997;19(3):406-13.
15. Anderson MA, Helms LB, Black S, Myers DK. A rural perspective on home care communication about elderly patients after hospital discharge. Western journal of nursing research. 2000;22(2):225-43.
16. Anderson MA, Hill PD. Psychometric Properties of the Referral Data Inventory. Applied Nursing Research. 1994;7(3):151-7.
17. Beard H. Does intermediate care minimize relocation stress for patients leaving the ICU? Nursing in critical care. 2005;10(6):272-8.
18. Bennett RE, Tuttle M, May K, Harvell J, Cole EA. Health Information Exchange in Post-Acute and Long-Term Care Case Study Findings: Final Report: Office of the Assistant Secretary for Planning and Evaluation Contract No.: Report.
19. Biddiss E, Chau T. The roles of predisposing characteristics, established need, and enabling resources on upper extremity prosthesis use and abandonment. Disability & Rehabilitation: Assistive Technology. 2007;2(2):71-84.
20. Bl C, Kb F, Pw A, Db W, Pj K, Jd D, et al. The Iowa Continuity of Care study: Background and methods. American journal of health-system pharmacy : AJHP : official journal of the American Society of Health-System Pharmacists2008. p. 1631-42.
21. Bodenheimer T. Coordinating care: a major (unreimbursed) task of primary care. Annals of Internal Medicine. 2007;147(10):730-1.
22. Bodenheimer T. Coordinating care--a perilous journey through the health care system. The New England journal of medicine. 2008;358(10):1064-71.
23. Bodenheimer T, Pham HH. Primary care: current problems and proposed solutions. Health affairs. 2010;29(5):799-805.
24. Bolton LB, Nelson M. CalNOC results 2002: links between staffing & patient perceptions of care... 35th Annual Communicating Nursing Research Conference/16th Annual WIN Assembly, "Health Disparities: Meeting the Challenge," held April 18-20, 2002, Palm Springs, California. Communicating nursing research. 2002;35(Journal Article):101-.
25. Boockvar KS, Livote EE, Goldstein N, Nebeker JR, Siu A, Fried T. Electronic health records and adverse drug events after patient transfer. Quality & Safety in Health Care. 2010;19(5):e16-e.
26. Br G, Md G, Rr F, Mt L, Dw G. The Washington Circle continuity of care performance measure: predictive validity with adolescents discharged from residential treatment. Journal of substance abuse treatment2010. p. 3-11.
27. Brankline AL, Coyle CM, Jencks KA, Mullegama A, O'Brien MW. Practical innovations: technology-assisted referrals. Social work in health care. 2009;48(8):768-76.
28. Brown JB, Stassen NA, Cheng JD, Sangosanya AT, Bankey PE, Gestring ML. Trauma center designation correlates with functional independence after severe but not moderate traumatic brain injury. Journal of Trauma. 2010;69(2):263-9.
29. Buurma H, Bouvy ML, De Smet PAG, Floor-Schreudering A, Leufkens HGM, Egberts ACG. Prevalence and determinants of pharmacy shopping behaviour. Journal of Clinical Pharmacy & Therapeutics. 2008;33(1):17-23.
30. C N, H S, A H, L B, D P, G T. Continuity of midwifery care and gestational weight gain in obese women: a randomised controlled trial. BMC public health2011. p. 174.
31. Carboni RM, Reppetto MA. Nursing records: an administrative and legal instrument for assistance [Portuguese]. Revista paulista de enfermagem. 2004;23(3):260-4.
32. Catania K, Askew T, Birkhimer D, Courtney LC, Hanes D, Lamprecht M, et al. From unit based to population focused: transforming the role of oncology clinical nurse specialists. Clinical Nurse Specialist: The Journal for Advanced Nursing Practice. 2012;26(2):103-6.
33. Center for Law and SP, Schumacher R, Hoffmann E. Continuity of Care: Research-Based Rationale2008 Contract No.: Report.
34. Ch O, S N, J N, M P, J H, B R, et al. The perception of medical professionals and medical students on the usefulness of an emergency medical card and a continuity of care report in enhancing continuity of care. International journal of medical informatics2011. p. 412-20.
35. Chariatte V, Berchtold A, Akré C, Michaud PA, Suris JC. Missed appointments in an outpatient clinic for adolescents, an approach to predict the risk of missing. Journal of Adolescent Health. 2008;43(1):38-45.
36. Chugg R. Managed networks and integrated children's services -- case study of Devon. Journal of Integrated Care. 2009;17(6):37-45.
37. Claflin N. Computerized interdisciplinary assessment. Journal for Healthcare Quality: Promoting Excellence in Healthcare. 2000;22(2):25-33.
38. Cyr M, Martens AC, Berbiche D, Perreault S, Blais L. Continuity of care in the ambulatory treatment of adolescents with asthma. Journal of Adolescent Health. 2006;39(6):926.e11-7.
39. D CM, H JS. Does continuity of care improve patient outcomes? (Provisional abstract). Journal of Family Practice2004. p. 974-80.
40. Da C, Ja W. Can continuity of care be improved? Results from a randomized pilot study. Ambulatory pediatrics : the official journal of the Ambulatory Pediatric Association2004. p. 336-9.
41. De Vliegher K, Paquay L, Vernieuwe S, Van Gansbeke H. The experience of home nurses with an electronic nursing health record. International nursing review. 2010;57(4):508-13.
42. Deese D, Stein M. Information systems & technology. The ultimate health care IT consumers: how nurses transform patient data into a powerful narrative of improved care [corrected] [published erratum appears in NURS ECON 2005 Jan-Feb;23(1):45]. Nursing economic$. 2004;22(6):336-41.
43. Dempsey C, Budzyna T, Madden S. Improve processes with perioperative EHR. Health Management Technology. 2009;30(10):32-3.
44. Dolin RH, Alschuler L, Boyer S, Beebe C, Behlen FM, Biron PV, et al. HL7 Clinical Document Architecture, Release 2. Journal of the American Medical Informatics Association : JAMIA. 2006;13(1):30-9.
45. Doyle D, Cornes M. Mainstreaming interprofessional partnerships in a metropolitan borough. Journal of Integrated Care. 2006;14(5):27-36.
46. Ea E. Continuity-of-care measures. Random assignment of patients to providers and the impact of utilization level. Medical care1990. p. 180-90.
47. Ejlertsson G, Berg S. Continuity-of-care measures. An analytic and empirical comparison. Medical care. 1984;22(3):231-9.
48. Estabrooks CA, Midodzi WK, Cummings GG, Ricker KL, Giovannetti P. The impact of hospital nursing characteristics on 30-day mortality. Nursing research. 2005;54(2):74-84.
49. F M, G V. Developing a virtual patient record using XML and web-based workflow technologies. International journal of medical informatics2003. p. 131-9.
50. Fernández, Codinach AB, Robles AV, Morcillo AR, Palacios NB, Villanueva GF, et al. Accessibility plan: can offer a higher quality for a lower cost? [Spanish]. Revista Rol de Enfermería. 2011;34(12):18-24.
51. Ferrauiola L, Bordonaro B. One-on-One with HUMC's CIO Lex Ferrauiola & CTO Ben Bordonaro. Hackensack University Medical Center's IT chiefs are taking on the ultimate task--a big to big conversion. Interview by Anthony Guerra. Healthcare Informatics. 2008;25(10):58-9.
52. Ferris N. Entry point. Health affairs. 2010;29(4):583-4.
53. Fitzgerald LR, Bauer M, Koch SH, King SJ. Hospital discharge: recommendations for performance improvement for family carers of people with dementia. Australian Health Review. 2011;35(3):364-70.
54. Floor-Schreudering A, De Smet PA, Buurma H, Egberts AC, Bouvy ML. Documentation quality in community pharmacy: completeness of electronic patient records after patients' first visits. Annals of Pharmacotherapy. 2009;43(11):1787-94.
55. Fogg JT. Interview with a quality leader: Dale W. Bratzler, DO, MPH on performance measures. Journal for Healthcare Quality: Promoting Excellence in Healthcare. 2010;32(2):24-8.
56. Fw S, Lp dW, Iw N, Hf C. Effects of transmural care on coordination and continuity of care. Patient education and counseling2000. p. 73-81.
57. G E, S B. Continuity of care in health care teams. A comparison of continuity measures and organisational solutions. Scandinavian Journal of Primary Health Care1985. p. 79-85.
58. G V, A K, C H, K C, L B, Ab S, et al. Patients report improvements in continuity of care when quality of life assessments are used routinely in oncology practice: secondary outcomes of a randomised controlled trial. European journal of cancer (Oxford, England : 1990)2010. p. 2381-8.
59. G W, J K. Continuity of care for older patients in family practice: how important is it? (Structured abstract). Canadian Family Physician2006. p. 754-5.
60. Gabriel BA. Telemedicine then and now: the legacy of Dorothy McHugh. Caring. 2004;23(7):20.
61. Gimpel N, Marcee A, Kennedy K, Walton J, Lee S, DeHaven MJ. Patient Perceptions of a Community-Based Care Coordination System. Health Promotion Practice. 2010;11(2):173-81.
62. Govindarajan P, Larkin GL, Rhodes KV, Piazza G, Byczkowski TL, Edwards M, et al. Patient-centered Integrated Networks of Emergency Care: Consensus-based Recommendations and Future Research Priorities... 2010 Academic Emergency Medicine (AEM) Consensus Conference, “Beyond Regionalization: Integrated Networks of Emergency Care”, June 2, 2010, Phoenix, Arizona. Academic Emergency Medicine. 2010;17(12):1322-9.
63. Green DL, Boonstra JA, Bober MA. Use of a codified medication process for documentation of home medications. Journal of the American Medical Informatics Association. 2010;17(5):608-12.
64. Grimes TC, Duggan CA, Delaney TP, Graham IM, Conlon KC, Deasy E, et al. Medication details documented on hospital discharge: cross-sectional observational study of factors associated with medication non-reconciliation. British journal of clinical pharmacology. 2011;71(3):449-57.
65. Groeschen HM. Electronic system improves medication reconciliation rates. American Journal of Health-System Pharmacy. 2007;64(18):1894-.
66. Guice KS. Why Are Effective Handoffs Critically Important: Non Paid ADAS Contract No.: Report.
67. Haggerty JL, Reid RJ, Freeman GK, Starfield BH, Adair CE, McKendry R. Continuity of care: a multidisciplinary review. British medical journal. 2003;327(7425):1219-21.
68. Hagland M. Island in the stream? CIOs are approaching surgery as either a standalone operation or a link in the EMR-enabled continuum of care. Healthcare Informatics. 2008;25(11):43-5.
69. Hansen LO, Strater A, Smith L, Lee J, Press R, Ward N, et al. Hospital discharge documentation and risk of rehospitalisation. Bmj Quality & Safety. 2011;20(9):773-8.
70. Hansen LO, Young RS, Hinami K, Leung A, Williams MV. Interventions to Reduce 30-Day Rehospitalization: A Systematic Review. Annals of Internal Medicine. 2011;155(8):520-U94.
71. Haukoos JS, White DA, Lyons MS, Hopkins E, Calderon Y, Kalish B, et al. Operational methods of HIV testing in emergency departments: a systematic review. Annals of Emergency Medicine. 2011;58(1):S96-103.
72. Hayrinen K, Lammintakanen J, Saranto K. Evaluation of electronic nursing documentation-Nursing process model and standardized terminologies as keys to visible and transparent nursing. International journal of medical informatics. 2010;79(8):554-64.
73. Helleso R, Lorensen M. Inter-organizational continuity of care and the electronic patient record: A concept development. International journal of nursing studies. 2005;42(7):807-22.
74. Hellesø R, Lorensen M, Sorensen L. Challenging the information gap – the patients transfer from hospital to home health care. Improving Patient Safety with Technology. 2004;73(7–8):569-80.
75. Helleso R, Sorensen L, Lorensen M. Nurses' information management across complex health care organizations. International journal of medical informatics. 2005;74(11-12):960-72.
76. Herndon L, Bones C, Kurapati S, Rutherford P, Vecchioni N. How-to Guide: Improving Transitions from the Hospital to Skilled Nursing Facilities to Reduce Avoidable Rehospitalizations. : Institute for Healthcare Improvement; . Available at www.IHI.org. Cambridge, MA: IHI, Institute for Healthcare Improvement2012 Contract No.: Report.
77. Hsieh YH, Jung JJ, Shahan JB, Pollack HA, Hairston HS, Moring-Parris D, et al. Outcomes and cost analysis of 3 operational models for rapid HIV testing services in an academic inner-city emergency department. Annals of Emergency Medicine. 2011;58(1):S133-9.
78. Indyk L, Indyk D. Collecting data along the continuum of prevention and care: a continuous quality improvement approach. Social work in health care. 2006;42(3):47-60.
79. J M, A H, S D, C D, Jr M. A practical clinical trial of coordinated care management to treat substance use disorders among public assistance beneficiaries. Journal of Consulting and Clinical Psychology2009. p. 257-69.
80. J M, N D, P T, J V, L PdC, E B. Rapid two-stage emergency department intervention for seniors: impact on continuity of care. Academic emergency medicine : official journal of the Society for Academic Emergency Medicine2003. p. 233-43.
81. Jones L, Doebbeling CC. Primary care utilization patterns before and after lung cancer diagnosis. European Journal of Cancer Care. 2009;18(2):165-73.
82. Key-Solle M, Paulk E, Bradford K, Skinner AC, Lewis MC, Shomaker K. Improving the Quality of Discharge Communication With an Educational Intervention. Pediatrics. 2010;126(4):734-9.
83. Khan S, Khan A, Feyz M. Decreased length of stay, cost savings and descriptive findings of enhanced patient care resulting from an integrated traumatic brain injury programme. Brain Injury. 2002;16(6):537-54.
84. Kleinpeter MA. Wildcard. End-stage renal disease use in hurricane-prone areas: should nephrologists increase the utilization of peritoneal dialysis? Advances in Chronic Kidney Disease. 2007;14(1):100-4.
85. Klimek JS. MTM services standards improve patient safety. Health Management Technology. 2009;30(7):20-1.
86. Kramer JS, Hopkins PJ, Rosendale JC, Garrelts JC, Hale LS, Nester TM, et al. Implementation of an electronic system for medication reconciliation [corrected] [published erratum appears in AM J HEALTH SYST PHARM AJHP 2007 Apr 1;64(7):684]. American Journal of Health-System Pharmacy. 2007;64(4):404-22.
87. Kuo GM. Medication errors in community/ambulatory care: incidence and reduction strategies. Journal of Pharmaceutical Finance, Economics & Policy. 2006;15(3):43-136.
88. Kuusisto A, Asikainen P, Lukka H, Tanttu K. Experiences with the Electronic Nursing Discharge Summary. Studies in Health Technology and Informatics. 2009;146(Journal Article):226-30.
89. L D, R G, V I, A L, C B, J K, et al. Use of a critical time intervention to promote continuity of care after psychiatric inpatient hospitalization. Psychiatric services (Washington, DC)2009. p. 451-8.
90. L P, F S, S V, E M, Pa B, M B. Evaluation of short-term effectiveness of the disease management program "Di.Pro.Di." on continuity of care of patients with congestive heart failure. Journal of the American Geriatrics Society2010. p. 1603-4.
91. Lee P. Ideal principles and characteristics of a fail-safe medication-use system. American Journal of Health-System Pharmacy. 2002;59(4):369-71.
92. Levanda M. Implementing a medication reconciliation process in a community hospital. American Journal of Health-System Pharmacy. 2007;64(13):1372.
93. Levine C, Halper D, Peist A, Gould DA. Bridging Troubled Waters: Family Caregivers, Transitions, And Long-Term Care. Health affairs. 2010;29(1):116-24.
94. Litvin CB. In the dark -- the case for electronic health records. New England Journal of Medicine. 2007;356(24):2454-5.
95. Lou WW. Statistical Study on Measures of Continuity of Care: National Center for Health Services Research Contract No.: Report.
96. Lubelchek RJ, Kroc KA, Levine DL, Beavis KG, Roberts RR. Routine, rapid HIV testing of medicine service admissions in the emergency department. Annals of Emergency Medicine. 2011;58(1):S65-70.
97. M A, E L, R L, X X, A C, N S, et al. Impact of a standardized communication system on continuity of care between family physicians and the emergency department. CJEM : Canadian journal of emergency medical care = JCMU : journal canadien de soins médicaux d'urgence2007. p. 79-86.
98. M C, Q Y, A S, D J, Kc C. Continuity of care and health decline associated with a hip fracture. Journal of Aging & Health2002. p. 385-98.
99. M D, M M, M B, G D, J àC, A dB, et al. A real-life observational study of the effectiveness of FACT in a Dutch mental health region. BMC psychiatry2008. p. 93.
100. M K, L J, O M, M R, A R, R W, et al. Development and pilot evaluation of a complex intervention to improve experienced continuity of care in patients with cancer. British journal of cancer2009. p. 274-80.
101. Macy RJ, Johns N. Aftercare Services for International Sex Trafficking Survivors: Informing U.S. Service and Program Development in an Emerging Practice Area. Trauma, Violence & Abuse. 2011;12(2):87-98.
102. Mangione-Smith R, Chung P, McGlynn EA, Schneider E. Conceptual Framework for Care Coordination/ Measures for Children with Complex Needs (COE4CCN). Conceptural Framework for Care Coordination/Fragmentation in the context of the Patient-Centered Medical Home for Children with Complex Needs: Agency for Healthcare Research and Quality (AHRQ) Contract No.: Report.
103. Manias E, Gerdtz MF, Weiland TJ, Collins M. Medication use across transition points from the emergency department: identifying factors associated with medication discrepancies. Annals of Pharmacotherapy. 2009;43(11):1755-64.
104. McDonald KM, Schultz E, Chapman T, Davies S, Pineda N, Lonhart J, et al. Prospects for Care Coordination Measurement Using Electronic Data Sources (Prepared by Stanford University under subcontract to Battelle on Contract No. 290-04-0020--AHRQ SQI-II.). Rockville, MD: Agency for Healthcare Research and Quality.2012 Contract No.: Report.
105. McHugh C, Robinson A, Chesters J. Health promoting health services: a review of the evidence. Health promotion international. 2010;25(2):230-7.
106. Me OC, Bs M, D G. Effect of open access scheduling on missed appointments, immunizations, and continuity of care for infant well-child care visits. Archives of Pediatrics & Adolescent Medicine2006. p. 889-93.
107. Merritt MK, Procter N. Conceptualising the functional role of mental health consultation-liaison nurse in multi-morbidity, using Peplau's nursing theory. Contemporary Nurse: A Journal for the Australian Nursing Profession. 2010;34(2):158-66.
108. Michèle A, Anik G, René V, I FM, Arminée K. Interventions to improve continuity of care in the follow-up of patients with cancer. Cochrane Database of Systematic Reviews2009.
109. Michels RD, Meisel SB. Program using pharmacy technicians to obtain medication histories. American Journal of Health-System Pharmacy. 2003;60(19):1982-.
110. Mira RC, D VG, J GS, Edward W, ThM vEJ, Jj AW. Interventions to improve the management of diabetes mellitus in primary care, outpatient and community settings. Cochrane Database of Systematic Reviews2000.
111. Mirtallo JM, Hawksworth K, Payne B. A nutrition support service web application to manage patients receiving parenteral nutrition. Nutrition in Clinical Practice. 2009;24(4):447-58.
112. Mj H, Dm S, Cr S, S S. Reorganization of a medical house staff (firm system): its effect on accessibility and continuity of care. Journal of Community Health1980. p. 6-17.
113. Moss J, Xiao Y. Improving operating room coordination: communication pattern assessment. Journal of Nursing Administration. 2004;34(2):93-100.
114. Motamedi SM, Posadas-Calleja J, Straus S, Bates DW, Lorenzetti DL, Baylis B, et al. The efficacy of computer-enabled discharge communication interventions: a systematic review. Bmj Quality & Safety. 2011;20(5):403-15.
115. Murff HJ. Medication errors in hospital care: incidence and reduction strategies. Journal of Pharmaceutical Finance, Economics & Policy. 2006;15(4):5-71.
116. Na A, J H, Sp H, Jm OB, Gs P, M R, et al. Continuity of care in intensive care units: a cluster-randomized trial of intensivist staffing. American journal of respiratory and critical care medicine2011. p. 803-8.
117. National Center for PA, Herrick D. Retail Clinics: Convenient and Affordable Care2010 Contract No.: Report.
118. Nauert RC. The new millennium: health care evolution in the 21st century. Journal of health care finance. 2000;26(3):1-14.
119. Neyhart CD, McCoy L, Rodegast B, Gilet CA, Roberts C, Downes K. A new nursing model for the care of patients with chronic kidney disease: the UNC Kidney Center Nephrology Nursing Initiative. Nephrology Nursing Journal. 2010;37(2):121-31.
120. Odle TG. From prevention to survivorship: the cancer care continuum. Radiation Therapist. 2008;17(2):89-109.
121. Olsom DM, Bettger JP, Alexander KP, Kendrick AS, Irvine JR. Transition of Care for Acute Stroke and Myocardial Infarction Patients: From Hospitalization to Rehabilitation, Recovery, and Secondary Prevention. Evidence Reprot/Technology Assessment, Number 202: National Center for Health Services Research Contract No.: Report.
122. O'Rourke J. Q-and-A: the perfect fit... Sheree L. Porter. Rehab Management: The Interdisciplinary Journal of Rehabilitation. 2010;23(7):22-3.
123. Pap OH. Examining the discharge planning process: an evaluation of two models in an acute care community hospital. Not found1984.
124. Pearson A, Porritt KA, Doran D, Vincent L, Craig D, Tucker D, et al. A comprehensive systematic review of evidence on the structure, process, characteristics and composition of a nursing team that fosters a healthy work environment. International Journal of Evidence-Based Healthcare. 2006;4(2):118-59.
125. Reid R, Haggerty J, Reid JR. Defusing the Confusion: Concepts and measures of cointinuity of healthcare: Final Report 2002. Ottawa, Canada: Canadian Health Services Research Foundation2002 Contract No.: Report.
126. Rogers J, Curtis P. The concept and measurement of continuity in primary care. American Journal of Public Health. 1980;70(2):122-7.
127. Rosa, Rosânegela. Discharge from neonatal intensive care unit and health care continuity: a bibliographic study [Portuguese]. Revista Mineira de Enfermagem. 2010;14(1):103-9.
128. Salmoni A. Continuity of Care for Community Dwelling Seniors: Canadian Health Science Research Foundation2003 Contract No.: Report.
129. Santos S, Murphy G, Baxter K, Robinson KM. Organisational factors affecting the quality of hospital clinical coding. Health Information Management Journal. 2008;37(1):25-37.
130. Schadewaldt V, Schultz T. Nurse-led clinics as an effective service for cardiac patients: results from a systematic review. International Journal of Evidence-Based Healthcare. 2011;9(3):199-214.
131. Schmitt BP, Hoffstadter L, Greider S, Thomas K, Calubaquib E, Montgomery E, et al. Using patient traffic control to reduce treatment delays for high-risk patients at a VA hospital. Joint Commission Journal on Quality & Patient Safety. 2008;34(3):147-53.
132. Schrantz SJ, Babcock CA, Theodosis C, Brown S, Mercer S, Pillow MT, et al. A targeted, conventional assay, emergency department HIV testing program integrated with existing clinical procedures. Annals of Emergency Medicine. 2011;58(1):S85-8.
133. Sevin C, Evdokimoff M, Sobolewski S, Taylor J, Rutherford P, Coleman EA. How-to Guide: Improving Transitions from the Hospital to Home Health Care to Reduce Avoidable Rehospitalizations. Cambridge, MA: Institute for Healthcare Improvement2012 Contract No.: Report.
134. Shorr GI, Nutting PA. Population-Based Assessment of the Continuity of Ambulatory Care: Bureau of Health Planning and Resources Development Health Research Administration Contract No.: Report.
135. Showalter JW, Rafferty CM, Swallow NA, DaSilva KO, Chuang CH. Effect of Standardized Electronic Discharge Instructions on Post-Discharge Hospital Utilization. Journal of General Internal Medicine. 2011;26(7):718-23.
136. Solovy A. Connecting all your docs: hospitals subsidize physicians---employed and independent---to make electronic medical records more dynamic. H&HN: Hospitals & Health Networks. 2009;83(9):28.
137. Sparbel KJH, Anderson MA. Integrated literature review of continuity of care: Part 1, conceptual issues. Journal of Nursing Scholarship. 2000;32(1):17-24.
138. Sparbel KJH, Anderson MA. A continuity of care integrated literature review, part 2: Methodological issues. Journal of Nursing Scholarship. 2000;32(2):131-5.
139. Stein M, Dixon J. Young people leaving care in Scotland. European Journal of Social Work. 2006;9(4):407-23.
140. Stewart J. Sideways. Practical Neurology. 2007;7(3):142-4.
141. Stuebe A, Ecker J, Bates DW, Zera C, Bentley-Lewis R, Seely E. Barriers to follow-up for women with a history of gestational diabetes. American Journal of Perinatology. 2010;27(9):705-10.
142. Taylor M. Working through the frustrations of clinical integration. H&HN: Hospitals & Health Networks. 2008;82(1):34-40.
143. Thompson-Moore N, Liebl MG. Health care system vulnerabilities: Understanding the root causes of patient harm. American Journal of Health-System Pharmacy. 2012;69(5):431-6.
144. Toby G, Frode F, Ivar K, Matthew S, Brenda L, Antonio G, et al. Capitation, salary, fee-for-service and mixed systems of payment: effects on the behaviour of primary care physicians. Cochrane Database of Systematic Reviews2000.
145. Varkey P, Cunningham J, O'Meara J, Bonacci R, Desai N, Sheeler R. Multidisciplinary approach to inpatient medication reconciliation in an academic setting. American Journal of Health-System Pharmacy. 2007;64(8):850-4.
146. Wagner L. New technologies enhance patient safety. Provider. 2004;30(3):20.
147. Waibel S, Henao D, Aller M-B, Vargas I, Vazquez M-L. What do we know about patients' perceptions of continuity of care? A meta-synthesis of qualitative studies. International Journal for Quality in Health Care. 2012;24(1):39-48.
148. Wakefield DS, Ward M, Miller T, Ohsfeldt R, Jaana M, Lei Y, et al. Intensive care unit utilization and interhospital transfers as potential indicators of rural hospital quality. Journal of Rural Health. 2004;20(4):394-400.
149. Walker JD, Morris K, Frood J. CIHI survey: alternative level of care in Canada: a summary. Healthcare Quarterly. 2009;12(2):21-3.
150. Waneka R, Spetz J. Hospital Information Technology Systems' Impact on Nurses and Nursing Care. Journal of Nursing Administration. 2010;40(12):509-14.
151. Wang N, Hailey D, Yu P. Quality of nursing documentation and approaches to its evaluation: a mixed-method systematic review. Journal of Advanced Nursing. 2011;67(9):1858-74.
152. Waters D, Picone D, Cooke H, Dyer K, Brodie P, Middleton S. Midwifery-led care: finding evidence for an antenatal model. Australian Midwifery. 2004;17(2):16-20.
153. Weiner M, Quwatli Z, Perkins AJ, Lewis JN, Callahan CM. Limitation of a single clinical data source for measuring physicians' performance on quality indicators. Journal of the American Geriatrics Society. 2006;54(8):1256-60.
154. White DA, Scribner AN, Vahidnia F, Dideum PJ, Gordon DM, Frazee BW, et al. HIV screening in an urban emergency department: comparison of screening using an opt-in versus an opt-out approach. Annals of Emergency Medicine. 2011;58(1):S89-95.
155. Wilson JW, Marshall WF, Estes LL. Detecting Delayed Microbiology Results After Hospital Discharge: Improving Patient Safety Through an Automated Medical Informatics Tool. Mayo Clinic proceedings. 2011;86(12):1181-5.
156. Wolfe DL, Hsieh JTC, Curt A, Teasell RW. Neurological and functional outcomes associated with SCI rehabilitation. Topics in Spinal Cord Injury Rehabilitation. 2007;13(1):11-31.
